# Plant-on-chip: Core morphogenesis processes in the tiny plant *Wolffia australiana*

**DOI:** 10.1093/pnasnexus/pgad141

**Published:** 2023-04-19

**Authors:** Feng Li, Jing-Jing Yang, Zong-Yi Sun, Lei Wang, Le-Yao Qi, Sina A, Yi-Qun Liu, Hong-Mei Zhang, Lei-Fan Dang, Shu-Jing Wang, Chun-Xiong Luo, Wei-Feng Nian, Seth O’Conner, Long-Zhen Ju, Wei-Peng Quan, Xiao-Kang Li, Chao Wang, De-Peng Wang, Han-Li You, Zhu-Kuan Cheng, Jia Yan, Fu-Chou Tang, De-Chang Yang, Chu-Wei Xia, Ge Gao, Yan Wang, Bao-Cai Zhang, Yi-Hua Zhou, Xing Guo, Sun-Huan Xiang, Huan Liu, Tian-Bo Peng, Xiao-Dong Su, Yong Chen, Qi Ouyang, Dong-Hui Wang, Da-Ming Zhang, Zhi-Hong Xu, Hong-Wei Hou, Shu-Nong Bai, Ling Li

**Affiliations:** The High School Affiliated to Renmin University of China, Beijing 100080, China; Center of Quantitative Biology, Peking University, Beijing 100871, China; State Key Laboratory of Protein & Plant Gene Research, Peking University, Beijing 100871, China; College of Life Sciences, Peking University, Beijing 100871, China; Institute of Hydrobiology, Chinese Academy of Sciences, Wuhan 430072, China; GrandOmics Biosciences Ltd., Wuhan 430076, China; Department of Biological Sciences, Mississippi State University, Mississippi State, MS 39762, USA; The High School Affiliated to Renmin University of China, Beijing 100080, China; The High School Affiliated to Renmin University of China, Beijing 100080, China; College of Life Sciences, Peking University, Beijing 100871, China; College of Life Sciences, Peking University, Beijing 100871, China; College of Life Sciences, Peking University, Beijing 100871, China; Center of Quantitative Biology, Peking University, Beijing 100871, China; Center of Quantitative Biology, Peking University, Beijing 100871, China; The High School Affiliated to Renmin University of China, Beijing 100080, China; Department of Biological Sciences, Mississippi State University, Mississippi State, MS 39762, USA; GrandOmics Biosciences Ltd., Wuhan 430076, China; GrandOmics Biosciences Ltd., Wuhan 430076, China; GrandOmics Biosciences Ltd., Wuhan 430076, China; GrandOmics Biosciences Ltd., Wuhan 430076, China; GrandOmics Biosciences Ltd., Wuhan 430076, China; Key Laboratory of Plant Functional Genomics of the Ministry of Education, Jiangsu Co-Innovation Center for Modern Production Technology of Grain Crops, Yangzhou University, Yangzhou 225009, China; Key Laboratory of Plant Functional Genomics of the Ministry of Education, Jiangsu Co-Innovation Center for Modern Production Technology of Grain Crops, Yangzhou University, Yangzhou 225009, China; College of Life Sciences, Peking University, Beijing 100871, China; College of Life Sciences, Peking University, Beijing 100871, China; State Key Laboratory of Protein & Plant Gene Research, Peking University, Beijing 100871, China; College of Life Sciences, Peking University, Beijing 100871, China; Biomedical Pioneering Innovative Center (BIOPIC) and Beijing Advanced Innovation Center for Genomics (ICG), Beijing 100871, China; Center for Bioinformatics (CBI), Peking University, Beijing 100871, China; State Key Laboratory of Protein & Plant Gene Research, Peking University, Beijing 100871, China; College of Life Sciences, Peking University, Beijing 100871, China; Biomedical Pioneering Innovative Center (BIOPIC) and Beijing Advanced Innovation Center for Genomics (ICG), Beijing 100871, China; Center for Bioinformatics (CBI), Peking University, Beijing 100871, China; State Key Laboratory of Protein & Plant Gene Research, Peking University, Beijing 100871, China; College of Life Sciences, Peking University, Beijing 100871, China; Biomedical Pioneering Innovative Center (BIOPIC) and Beijing Advanced Innovation Center for Genomics (ICG), Beijing 100871, China; Center for Bioinformatics (CBI), Peking University, Beijing 100871, China; Key Laboratory of Plant Functional Genomics of the Ministry of Education, Jiangsu Co-Innovation Center for Modern Production Technology of Grain Crops, Yangzhou University, Yangzhou 225009, China; Key Laboratory of Plant Functional Genomics of the Ministry of Education, Jiangsu Co-Innovation Center for Modern Production Technology of Grain Crops, Yangzhou University, Yangzhou 225009, China; Key Laboratory of Plant Functional Genomics of the Ministry of Education, Jiangsu Co-Innovation Center for Modern Production Technology of Grain Crops, Yangzhou University, Yangzhou 225009, China; State Key Laboratory of Agricultural Genomics, BGI-Shenzhen, Shenzhen 518083, China; State Key Laboratory of Agricultural Genomics, BGI-Shenzhen, Shenzhen 518083, China; State Key Laboratory of Agricultural Genomics, BGI-Shenzhen, Shenzhen 518083, China; State Key Laboratory of Protein & Plant Gene Research, Peking University, Beijing 100871, China; College of Life Sciences, Peking University, Beijing 100871, China; State Key Laboratory of Protein & Plant Gene Research, Peking University, Beijing 100871, China; College of Life Sciences, Peking University, Beijing 100871, China; PASTEUR, Département de chimie, École normale supérieure, PSL University, Sorbonne Université, CNRS, 24 rue Lhomond, Paris 75005, France; Center of Quantitative Biology, Peking University, Beijing 100871, China; School of Physics, Peking University, Beijing 100871, China; State Key Laboratory of Protein & Plant Gene Research, Peking University, Beijing 100871, China; College of Life Sciences, Peking University, Beijing 100871, China; Institute of Botany, Chinese Academy of Sciences, Beijing 100093, China; State Key Laboratory of Protein & Plant Gene Research, Peking University, Beijing 100871, China; College of Life Sciences, Peking University, Beijing 100871, China; Institute of Hydrobiology, Chinese Academy of Sciences, Wuhan 430072, China; Center of Quantitative Biology, Peking University, Beijing 100871, China; State Key Laboratory of Protein & Plant Gene Research, Peking University, Beijing 100871, China; College of Life Sciences, Peking University, Beijing 100871, China; Department of Biological Sciences, Mississippi State University, Mississippi State, MS 39762, USA

**Keywords:** *Wolffia australiana*, high-quality genome, morphogenesis, plant-on-chip

## Abstract

A plant can be thought of as a colony comprising numerous growth buds, each developing to its own rhythm. Such lack of synchrony impedes efforts to describe core principles of plant morphogenesis, dissect the underlying mechanisms, and identify regulators. Here, we use the minimalist known angiosperm to overcome this challenge and provide a model system for plant morphogenesis. We present a detailed morphological description of the monocot *Wolffia australiana*, as well as high-quality genome information. Further, we developed the plant-on-chip culture system and demonstrate the application of advanced technologies such as single-nucleus RNA-sequencing, protein structure prediction, and gene editing. We provide proof-of-concept examples that illustrate how *W. australiana* can decipher the core regulatory mechanisms of plant morphogenesis.

**Significance Statement**

What is the core morphogenetic process in angiosperms, a plant like a tree indeterminately growing or a bud sequentially generating limited types of organs? *Wolffia australiana*, one of the smallest angiosperms in the world, may help to make a distinction. Wolffia plantlet constitutes of only three organs that are indispensable to complete life cycle: one leaf, one stamen, and one gynoecium. Before the growth tip is induced to flower, it keeps branching from the leaf axil, and the branches separate from the main plantlet. Here, we present a high-quality genome of *W. australiana*, detailed morphological description, a plant-on-chip cultural system, and some principle-proof experiments, demonstrating that *W. australiana* is a promising model system for deciphering core developmental program in angiosperms.

## Introduction

What are the core morphogenetic processes required for a multicellular organism to complete its life cycle? For most species in the animal kingdom, embryogenesis plays such a core role, with all organs initiated at that stage. By contrast, in most species in the plant kingdom, organs and tissues are produced sequentially. Plant development starts, like that of animals, with the formation of a zygote, whose number and types of organs are limited. However, plants then go on to produce an indeterminate number of organs such as leaves, roots, and stems before they produce spores and initiate the gametophyte for formation of haploid gametes. Also, in contrast to animals, during the process of completing the life cycle, in addition to the growth tip derived from the zygote, many plants can produce new growth tips in the axillary buds.

Waddington (1) pointed out that each apical meristem “gives rise to a whole new cycle of growth and development.” Since a plant comprises many branches derived from lateral growth, each plant may be seen as a colony of buds (or meristems) supporting growth, in essence rather comparable with the outer shell of a coral colony than to an individual worm, bird, or cat. Notably, unlike the broad synchronization of polyps inhabiting coral structures in their developmental process on an annual rhythm, plant branches or buds undergo their development independently of one another. For example, in the perennial plant alpine rock cress (*Arabis alpina*), a close relative of the model plant Arabidopsis (*Arabidopsis thaliana*), a subset of meristems will transition to their reproductive stage under conditions conducive to flowering, while other meristems will remain vegetative (2). Likewise, apple (*Malus domestica*) trees carry vegetative buds and floral buds in various developmental stages simultaneously to support vegetative growth and fruit harvest each year.

**Fig. 1.**
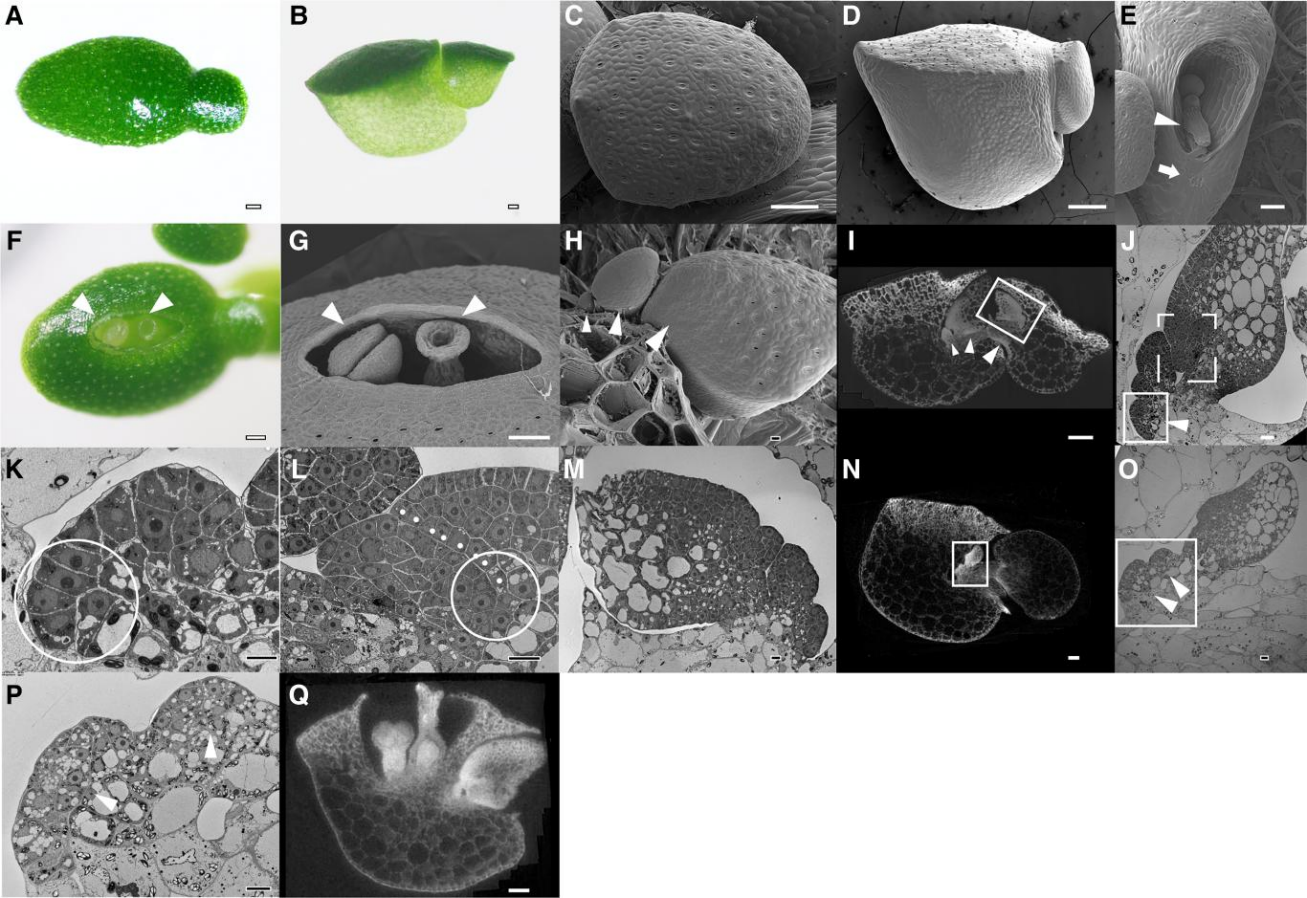
Morphological description of *W. australiana.* A) Top view of a *W. australiana* plantlet, as seen under a dissecting microscope; a branch is protruding to the right. B) Side view of a *W. australiana* plantlet, showing a boat-shaped leaf with dark green cells at the “deck” and light green cells at the “hull.” C) Top view of a *W. australiana* plantlet by cryo-SEM; note the presence of stomata. D) Side view of a *W. australiana* plantlet by cryo-SEM; no stomata were observed. E) View from the hole from which branches abscise out, showing the remaining petiole (arrowhead). A scar (arrow) forms on the boat-shaped leaf and indicates prior branch abscission. F) Top view of a *W. australiana* plantlet under a dissecting microscope, showing the stigma and stamen (arrowheads) protruding from the crack on the “deck.” G) Top view of the crack region of a *W. australiana* plantlet, as seen by cryo-SEM, showing a stigma (right, arrowhead) and a stamen (left, arrowhead). H) After peeling the deck, three young leaves (the biggest one has developed into a branch) are aligned sequentially, as indicated by three arrowheads. I) CT image showing the alignment of leaves (arrowheads) and how the biggest leaf has developed into a branch before abscission (the rectangle shows new leaves produced from the biggest leaf). J) TEM section of the leaf primordia and the region including the growth tip (rectangle with arrowhead). K) Zoomed-in region indicated by the solid rectangle shown in J). The circle highlights the cells with big nuclei and dense cytoplasm, possible including the growth tip cell(s). L) Zoomed-in region indicated by the dashed rectangle in J) with adjusted orientation. The dotted line indicates the border of the fast-growing region of the primordium leaf that overlaps with the slow-growing region. The circle indicates the junction where a growth tip of the primordium leaf might initiate de novo, which allows a primordium leaf to become a new branch. M) TEM section of the growth region of the branch (prepared with the CT sample, corresponding to the corresponding rectangle in Fig. 1I. N) CT image showing a region of the growth tip of a plantlet under flower induction conditions. The rectangle highlights the growth region for further observation. O) TEM section of the growth region (prepared with the CT sample, corresponding to the corresponding rectangle in Fig. 1N. Two bumps (arrowheads) arise from the innermost region of the cavity. P) Further enlargement of the region highlighted in Fig. 1O. Arrowheads indicate cells in the bumps that are morphologically different from those shown in Fig. 1K. Q) CT image showing a gynoecium (right) and a stamen (left) inside the plantlet, possibly derived from the two bumps observed in Fig. 1P. Bars = 100 *μ*m (A–G, I, N, Q) and 10 *μ*m (H, J–P).

While the morphogenetic strategy combining apical growth and branching rendered advantages for plants as sessile and photoautotrophic eukaryotes, it can present challenges in elucidating core morphogenetic processes due to a general lack of synchronization between meristems. One potential approach to address this challenge is to seek out plant species that naturally possess minimal morphological structures, such as essential organs and simple branching. By utilizing such species as experimental systems, it may be possible to avoid the problem of asynchrony of meristem development within a colony. We propose that *Wolffia australiana* may serve as such a model plant for investigating the core principles of plant morphogenesis, given its simplicity and accessibility (Fig. 1).

*Wolffia* is a genus of aquatic plants. It is the simplest and smallest known angiosperm in the world (3, 4). Each *W. australiana* plantlet consists of a single boat-like leaf with a diameter of 1 mm, with a few axillary buds wrapped around the base of the leaf, but no root. After the axillary buds grow, they separate (abscise) from the main plantlet. Under stress conditions, the plantlet produces one stamen and one gynoecium, which arise vertically upward from a hole generated in the center of the “deck” of the boat-like leaf. The stamen consists of two locules containing numerous pollen grains, while the gynoecium contains a single ovule (5, 6) (Fig. 1). Therefore, the *Wolffia* plantlet harbors a minimal set of core organs needed for an angiosperm to complete its life cycle and not much more.

What insights might be gleaned using the *Wolffia* plantlet as a model system? Work on the tiny angiosperm over the past 60 years provides some clues. For instance, the transition from vegetative branching to completing the floral organ morphogenesis only takes a few days, thus offering a unique opportunity to decipher the mechanisms of cell fate change along a precise spatiotemporal pattern (7, 8). *Wolffia* as a model system may thus unlock the developmental programs underlying plant morphogenesis.

The shoot apical meristem of *Wolffia* plantlets is also much simpler than that of other spermatophytes. While the shoot tip is made up of a cell cluster in gymnosperms and angiosperms, a single cell or a few cells are sufficient to carry out the functions of a growth tip, as in the haploid moss *Physcomitrium* (*Physcomitrella*) *patens*, or in the diploid pteridophytes such as *Selaginella* or *Adiantum*. Considering that the entire *Wolffia* plantlet is only 1 mm in diameter, and based on observations that revealed too few cells at the tip to organize into a higher-order structures as that seen in angiosperms (6, 9–13), the *Wolffia* growth tip holds the promise of a much simplified organization with no tunica-corpus structure that can produce leaves, axillary buds, and finally the stamen and gynoecium.

The root is a critical organ in terrestrial angiosperms, playing essential roles in anchoring the plant and nutrient uptake. In contrast to many duckweed species, such as *Lemna minor*, *Lemna gibba*, and *Spirodela polyrhiza*, which all have roots, all species in the *Wolffia* genus lack roots. The absence of roots in *Wolffia* is unlikely to represent a direct adaptation to an aquatic environment. By leveraging the wealth of information available on root development in other model plants, it is possible to generate testable hypotheses to shed light on the regulatory mechanisms underlying root development and its evolutionary origin.

Perhaps the most apparent advantage of *Wolffia* plantlets is that they only carry a single leaf, as each new branch will bud off as a separate plantlet. This growth pattern thus provides unique opportunities to continuously observe the same plantlet for its entire life cycle under a microscope.

The simplicity of the *Wolffia* genus and its duckweeds relatives has attracted much interest and has led to efforts to develop this tiny plant(let) into an experimental system (3–5, 14, 15). However, key tools are currently lacking to propel the *Wolffia* genus as a model system to investigate core principles of the plant life cycle. Here, we report our efforts in developing *W. australiana* as a model system. We sequenced the genome of this species, which will complement other genome sequences from this species recently published (16, 17), and set up a “plant-on-chip (PoC)” culture system with which to observe morphological characteristics, particularly in the diploid phase. We also exploited the genomic information we generated to analyze the possible mechanisms behind unique morphological traits seen in *W. australiana* such as the lack of a root or a vasculature and the fast transition from vegetative to reproductive growth. Furthermore, we demonstrated the feasibility of obtaining transgenic plantlets, single-nucleus RNA-sequencing (snRNA-seq) and protein structure prediction. We believe that this new PoC system will serve as a platform for dissection of core principles underlying plant morphogenesis in plant biology research.

## Results

### Morphological uniqueness: growth tip, branch, and floral organs

A typical plantlet (previously termed a “frond”) of *W. australiana* has one boat-shaped leaf with a branch (previously called a daughter frond) on the side (6) (Fig. 1A and B). Our detailed observations (below) revealed that frond is not the correct term for these structures. We therefore refer to them as plantlets and branches instead of fronds and daughter fronds, respectively, hereafter.

Close observations discovered that the deck part of the boat-shaped leaf is relatively dark green with smaller cells, while the hull part was relatively light green with larger cells (Fig. 1B). Using cryo-scanning electron microscopy (cryo-SEM), we observed stomata on the surface of the deck but not on the hull surface (Fig. 1C and D). We also noticed the presence of a scar near the hole from where the branched plantlets continuously protrude out (Fig. 1E), corresponding to plantlet abscission from the petiole linking the branching plantlet. The hole and scar provided clear markers to orientate a plantlet. Under inductive conditions (see below), we observed the emergence of a crack in the center of the deck, perpendicularly to the hole from which branching plantlets protrude. The floral organs, one stamen and one gynoecium, rose from the crack (Fig. 1F and G; video at https://www.wolffiapond.net, Username and Password: waus).

After peeling off the deck, we noticed three consecutive plantlets (on growing branches) of decreasing size (Fig. 1H). Such an alignment suggested that the branched plantlets are initiated sequentially inside the boat-shaped leaf. We confirmed this branch alignment by microcomputed tomography (CT) (Fig. 1I). This interpretation was also consistent with the observation that plantlets continuously protrude (see video at https://www.wolffiapond.net).

Where do branched plantlets arise? Focusing on the smallest primordium next to the inside surface of the boat-shaped leaf (Fig. 1J and K), we observed several cells with a big nucleus and a condensed cytoplasm, arranged in the innermost region. Such cellular characteristics are typically associated with meristematic cells, suggesting that these cells may constitute the growth tip in *W. australiana*. Indeed, the development of the growth tip from branched plantlets supported this hypothesis: following its initiation, the leaf primordium underwent asymmetric growth, with the outer portion of the primordium growing faster than the inner region. After the differentiation of the petiole (Fig. 1J), the fast-growing outer region protruded inward and overlapped with the slow-growing inner region (Fig. 1J and L). The space between the two regions then formed a cavity inside the boat-like leaf, after which point a new growth tip initiated a new branch at the innermost point of the cavity (Fig. 1L). We thus conclude that the functional growth tip in *W. australiana* is not a multicellular cluster with a tunica-corpus structure like that seen in angiosperms but only comprises one to a few cells that are induced during primordium differentiation. Such a growth tip organization repeated in each branch (compare Fig. 1M and J).

While all branched plantlets grew outward, the innermost region next to the inner surface of the boat-shaped leaf enlarged upon flower induction conditions, visible as two bumps (Fig. 1N to P). Compared with the specific orientation of floral organs (Fig. 1Q), these two bumps appeared to be the early stages of the stamen and the gynoecium primordia, respectively. Although we did not follow fertilization or seed development, we did observe the differentiation of floral organs (Fig. S1; https://www.wolffiapond.net).

### Genome sequence, assembly, and analysis

To aid in exploring the developmental innovations of the *Wolffia* genus, we performed whole-genome sequencing with a combination of strategies, including Nanopore PromethION (ultralong), Illumina NovaSeq 6000 and Hi-C reads, and Bionano to construct the reference genome for accession wa7733 (referred here as Waus). We generated 18.46 Gb of ultralong reads, 22.85 Gb of Illumina data, 253.08 Gb of Bionano data, and 36.55 Gb of Hi-C data (Table S1). To help with later genome annotation, we produced 202.46 Gb of data by transcriptome deep sequencing (RNA-seq). To survey the genome, we used 11.3 Gb of Illumina paired-end reads (out of 22.85 Gb), resulting in 8.3 Gb of clean reads after quality control steps and the removal of organellar and bacterial genomes. Based on these paired-end reads, we estimated the genome size to be 353,335,418 bp with a heterozygosity of <0.3% (Fig. S2 and Table S2), which indicates that the Waus genome is very homozygous.

**Fig. 2.**
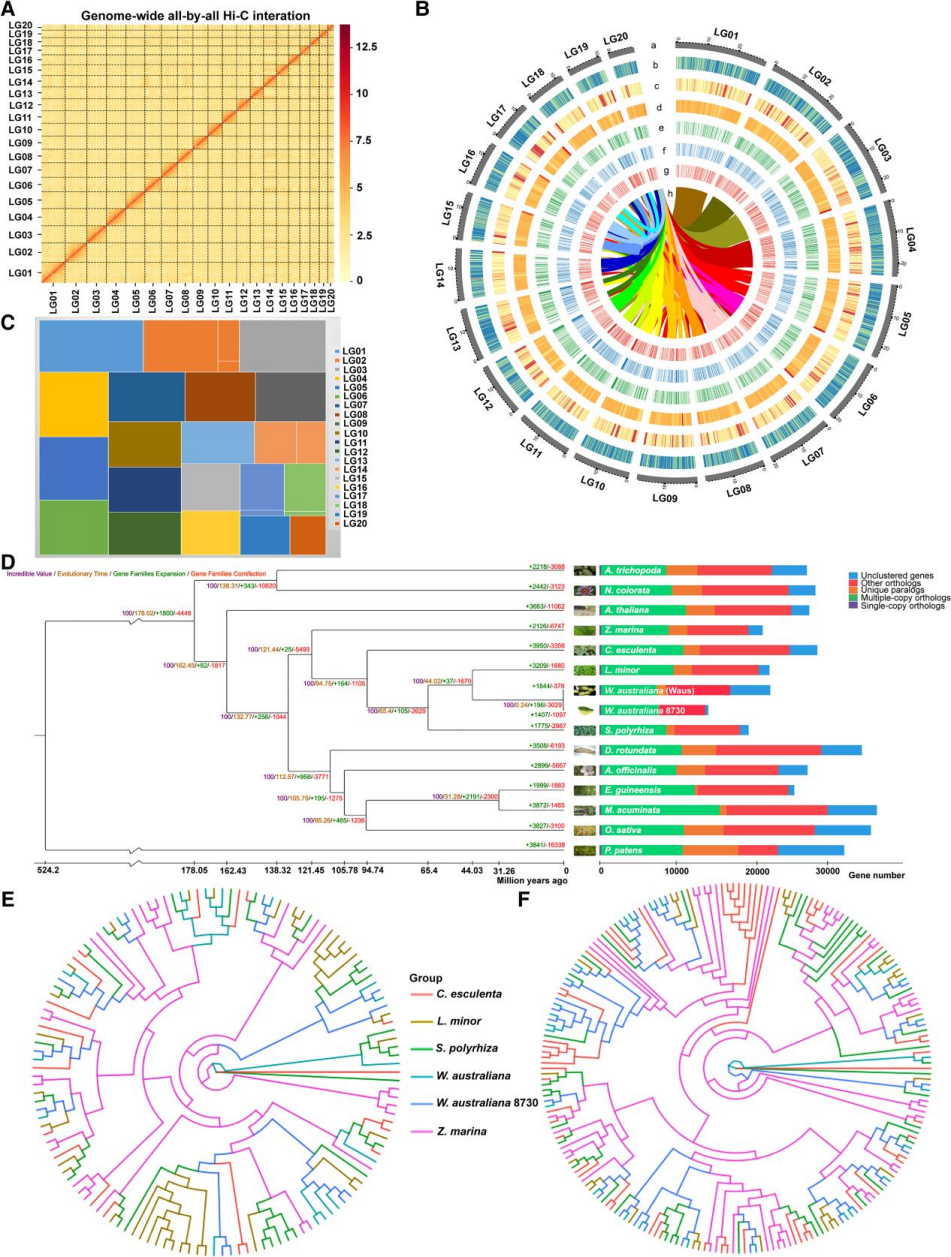
Genomic features of the *W. australiana* genome and gene family evolution in *W. australiana*. A) Hi-C interaction matrix for the 20 *W. australiana* pseudochromosomes. B) Circos plot of the *W. australiana* genomic features: (a) distribution of 20 chromosomes (each bar represents one chromosome, and the number represents the chromosome length); (b) gene density; (c) repeat sequence density; (d) GC contents; (e) gene density of control transcriptome; (f) gene density of flowered transcriptome; (g) gene density of induced transcriptome; and (h) synteny and distribution of genomic regions across the *W. australiana* genome. C) Treemap for contig length difference of 20 chromosomes. D) Phylogenetic analysis of *W. australiana* and other plants. The single-cell green alga *Chlamydomonas reinhardtii* was used as outgroup. The value on each node represents the divergence time in millions of years (mya). Nodes marked red are published fossil calibration time points. Numbers marked in green/red represent expansion/contraction numbers on each branch. Photos on the right show the corresponding species. E) *AGL* flowering-related genes. F) Root-related *SAUR* genes.

We used Nanopore reads to assemble the *W. australiana* genome into contigs. The G1 (genome version 1) contig length was 358.51 Mb, with an N50 size of 17.96 Mb. The G2 was 362.72 Mb (Table S3). We also identified 4,345,907 bp of sequences corresponding to the mitochondrial and chloroplast genomes or to other contaminants. We distinguished true genomic sequences from other contaminants based on their GC contents (Fig. S3A and B). The Bionano data were used to correct the G3 genome, and five gaps were produced, resulting in 26 sequences for G4 (size of 358.77 Mb) that we assembled into 20 pseudochromosomes (Fig. 2A to C and Table S4). The mapping rates of RNA-seq against the genome assembly were 95.1% (Table S5). Fluorescence in situ hybridization (FISH) analysis on prometaphase chromosomes confirmed 20 pairs of homologous chromosomes, representing 40 somatic chromosomes in the *W. australiana* genome (Fig. S4).

We also identified homozygous single-nucleotide polymorphism (SNP) sites and insertion/deletion (InDels) by comparing the genome sequence reported here with those from other *W. australiana* species, yielding 6,764 SNPs and 3,166 InDels with minimal support of at least five Illumina reads (Table S6). The contig N50 of our Waus genome was 18,579,918 bp compared with 251,357 bp for wa7733, 102,226 bp for wa8730, and 742,788 bp for wa8730 (three other published *W. australiana* genomes) (16, 17). Analysis of Benchmarking Universal Single-Copy Orthologs (BUSCO) demonstrated that our genome assembly is much more complete than previously sequenced and assembled *W. australiana* genomes, with BUSCO scores of 94.55% (Waus), 77.10% (wa7733), 80.29% (wa8730), and 87% (wa8730) (Tables 1 and S7).

**Table 1.** Statistics of *W. australiana* genome assembly comparison.

Name	Waus	wa7733 (16)	wa8730 (16)	wa8730 (17)
Total length (bp)	358,772,296	359,766,217	337,899,876	456,810,926
Contig number	25	2,578	5,250	1,757
Contig N50 (bp)	18,161,740	256,298	102,418	734,533
Longest contig (bp)	28,936,433	1,664,978	679,034	7,663,058
Scaffold number	20	2,578	5,250	1,508
Scaffold N50 (bp)	18,579,918	836,551	109,493	1,169,370
Longest scaffold (bp)	28,936,433	5,333,369	1,714,878	8,358,235
BUSCO	94.55%	77.10%	80.29%	∼87% (?)
Mapping TGS	99.50%	98%	96%	NA
Mapping NGS	99.80%	98%	95%	NA
Genome size (Mb)	358.8	359.8	337.9	∼456.
Protein-coding genes	22,484	15,312	14,324	22,293

### Genome annotation and phylogenetic analysis

The *W. australiana* genome Waus contained 227.8 Mb (or 63.5% of the genome) of repetitive regions, consisting of both transposable elements (TEs) and tandem repeats (TRs). TEs with long terminal repeats (LTRs) represented the majority (41.8%) of TEs, followed by DNA transposons (9.2%), long interspersed nuclear elements (3.9%), and miniature inverted-repeat transposable elements (*MITE*s) (1.1%) (Table S8). We predicted 1,658 noncoding RNAs (ncRNAs) for a total length of 237.2 kb, including 191 ribosomal RNAs, 867 ncRNAs, eight regulatory RNAs, and 592 transfer RNAs (Table S9). We also predicted 22,484 protein-coding genes with an average length per gene of 3,789 bp (Tables S10 and S11). Finally, we compared the predicted Waus protein sequences with biological databases, resulting in functional annotation for 69.5% of all genes. We also obtained support for 75.04% of all predicted gene models in RNA-seq samples (Table S10).

We determined the phylogenetic relationship between *W. australiana* and 14 other Viridiplantae species using a set of low-copy orthologous gene groups (Tables S11 and S12). Specifically, the phylogenetic results and fossil calibration time revealed that *W. australiana* 7733 diverged from *W. australiana* 8730 ∼0.24 million years ago (mya) (Fig. 2D and Table S13). We also compared the Waus genome with the genomes from other plant species to elucidate key genomic changes associated with adaptation to a small plant stature. We thus identified the expansion of 196 gene families and the contraction of 3,029 gene families in the cluster of *W. australiana* relative to plant species with larger body plans (Fig. 2D). For example, the *AGAMOUS-LIKE* (*AGL*) family involved in flowering typically clusters in 11 groups in flowering plants (Fig. 2E and Table S14) but only defined nine groups in the Waus genome, suggestive of an incomplete *AGL* family in *W. australiana.* Similarly, many of the root-related small auxin up-regulated RNA (*SAUR*) genes were missing in *W. australiana* (Fig. 2F and Table S14).

### Millifluidic setup for tracking plantlets

**Fig. 3.**
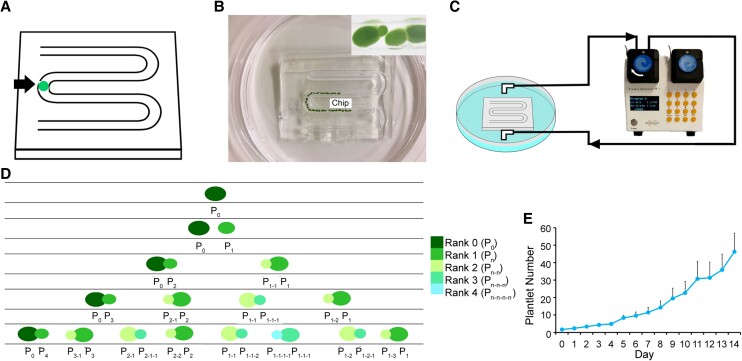
PoC culture platform. A) Representative millifluidic chip (detailed information in the Materials and methods section), showing a loaded plantlet. B) Abscised plantlets (former branch) line up along the channel. C) A peristaltic pump is connected to the chip to circulate liquid half-strength MS medium. D) Diagram of growth pattern; the branch ranks are indicated by different colors. E) Growth curve of cultured plantlets in the PoC in half-strength MS medium under short-day conditions at 26°C (*n* = 3).

To track the developmental processes of individual plantlets as they physically separate from one another, we designed a millifluidic chip system for plantlet culture. The chip design is relatively simple: we poured polydimethylsiloxane (PDSM) (using Momentive clear RTV615 potting and encapsulating compound in a 10:1 ratio) into molds to create 1-mm-wide channels (Fig. 3A). Each chip can hold over 100 plantlets and is sufficient to carry out most experiments.

To circulate the culture medium, we connected a peristaltic pump to the chip to help the liquid medium [half-strength Murashige and Skoog (MS)] flowing (Fig. 3B). Starting from a single plantlet with its first branch budding out along the long axis, the plantlets will line up along the channel (Fig. 3C). As the newly released (abscised) plantlet will also bud out of its own branch in the opposite orientation, the plantlets will align in a predictable order along the channel, as depicted in Fig. 3D. We designated the chip system “PoC.”

For the convenience of tracking morphogenesis and manipulating the growth conditions, we also designed a customized incubator controlled by a computer and connected to a digital camera (Fig. S5). All culture parameters can be programmed; plantlet growth can be monitored in an automated fashion. A typical growth curve under normal growth conditions (26°C, short-day photoperiod of 8-h light/16-h dark with 26.99 *μ*mol photons/m^2^/s) is shown in Fig. 3E. Under normal conditions, each plantlet can release a new plantlet about every 48 h (±12 h) and survive for about a month thereafter. We tested several flowering inductive conditions previously reported to induce flowering in *W. microscopia* (7, 8, 18) and successfully defined conditions that will induce flowering in *W. australiana* growing within the PoC (Fig. S6). We can therefore collect samples for morphological analyses (see above) and for the analysis of gene expression during flower development (see below).

### Examples of decipher morphogenetic events through genomic and gene network analyses

#### The rootless phenotype of *W. australiana*

A prominent morphological trait of *W. australiana* is its lack of roots. An attractive explanation would be that genes required for root development have been lost. However, we identified homologs for all known Arabidopsis genes involved in root development in the Waus genome (Table S14). The formation of an auxin gradient is crucial for root initiation and maintenance of all root types (19, 20). Intriguingly, in the region near the growth tip of *W. australiana*, cellular alignment required for an auxin gradient is hard to see (Fig. 1). These observations open up new opportunity to decipher the rootless phenotype and facilitate understanding of root morphogenesis in general. Furthermore, studying the absence of roots in *Wolffia* in comparison with its close relative duckweeds with roots could shed light on the physiological role of roots in aquatic plants and the mechanism of nutrient uptake. This comparative approach may offer insights into the evolutionary adaptation of plants to the aquatic environment and contribute to our understanding of the functional significance of roots in plant growth and development.

#### Lack of a vasculature

**Fig. 4.**
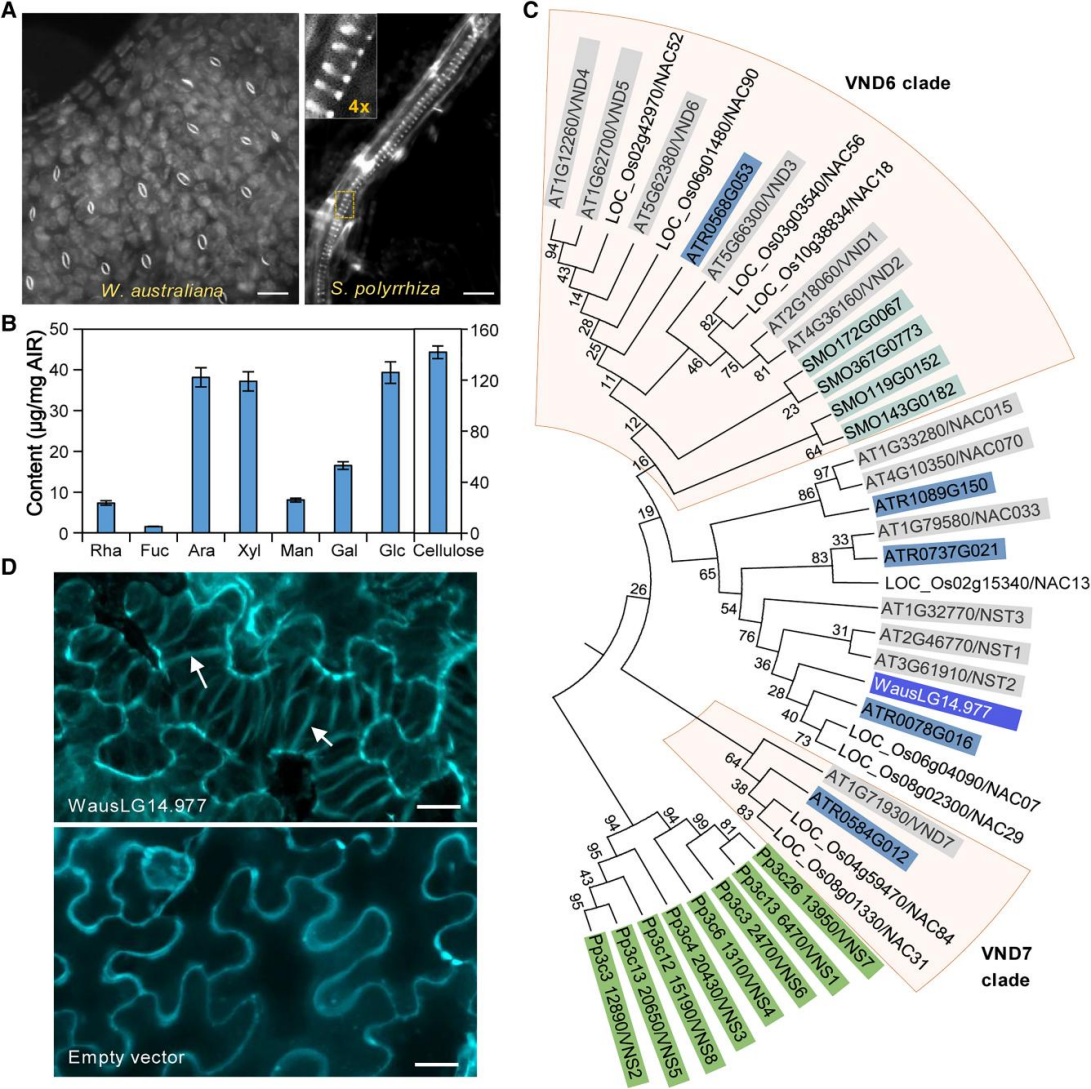
*W.* australiana lacks vasculature. A) Staining of *W. australiana* and *S. polyrhiza* plantlets with the cell wall dye Direct Red 23, revealing no SCW vascular structure in *W. australiana*, in contrast to the spiral-like xylem cells (inset) observed in the closely related duckweed *S. polyrhiza*. Bars = 80 *μ*m (left) and 20 *μ*m (right). B) Cell wall composition of *W. australiana* plantlets. All components are shown with the scale to the left, except cellulose (right scale). Bar charts represent the mean ± standard deviation (SD) of five biological replicates. C) Phylogenetic analysis of SCW–related NAC homologs in *W. australiana* and five representative genomes, indicating the absence of the VND homolog (boxed) in *W. australiana*. AT, *A. thaliana*; ATR, *A. trichopoda*; Os, *O. sativa*; Pp, *P. patens*; SMO, *S. moellendorffii*. D) Confocal images of *N. benthamiana* leaf epidermal cells transiently overexpressing WausLG14.977 or infiltrated with empty vector. Vessel-like cells were observed with WausLG14.977. Arrows indicate spiral SCW bands in a vessel-like cell. Bars = 20 *μ*m.

We observed no vascular tissue in *W. australiana*, in contrast to its close relative, duckweed *S. polyrhiza* (Fig. 4A). However, the cell wall composition of *W. australiana* is similar to that of aquatic plants harboring vascular bundles (21) (Fig. 4B). As xylem vessels usually possess thickened cell walls with specific patterns, referred to as the secondary cell wall (SCW), we looked for genes that are known to be involved in SCW formation in the Waus genome. We detected most SCW genes, indicating that cell wall biogenesis is likely intact in *W. australiana* (Table S15). However, in contrast to the 13 Arabidopsis and nine rice SCW–related *NAM-ATAF1,2-CUC2* (*NAC*) gene family members (Tables S15 and S16), which encode the upstream master regulators for SCW formation in vascular plants (22), we only identified one homolog in *W. australiana*. This gene, WausLG14.977, clustered separately from the groups defined by Arabidopsis VASCULAR-RELATED NAC DOMAIN PROTEIN6 (VND6, At5g62380) and VND7 (At1g71930) (Fig. 4C), two master NACs involved in xylem vessel differentiation (22).

To test the function of WausLG14.977, we transiently expressed the gene in *Nicotiana benthamiana* leaves. Based on the UV-excited fluorescent signals that derived from lignified SCW, we observed spiral SCW bands in the epidermal cells expressing WausLG14.977 but not in cells transiently infiltrated with the empty vector (Fig. 4D). The formation of vessel-like cells thus suggested that WausLG14.977 has the potential to be a functional VND-like regulator. This interpretation was further corroborated by the similarity in protein structure between the protein encoded by WausLG14.977 and Arabidopsis SECONDARY WALL-ASSOCIATED NAC DOMAIN PROTEIN1 (SND1, At1g32770), as predicted by RoseTTAFold (Fig. S7).

Taken together, while the single *NAC* family member WausLG14.977 appeared to be functional for SCW formation, loss of homologs from the VND6 and VND7 clades and/or the lack of downstream components essential for xylem vessel development may be responsible for the absence of vascular tissues in *W. australiana*.

#### Correlation between differential gene expression and flower development

**Fig. 5.**
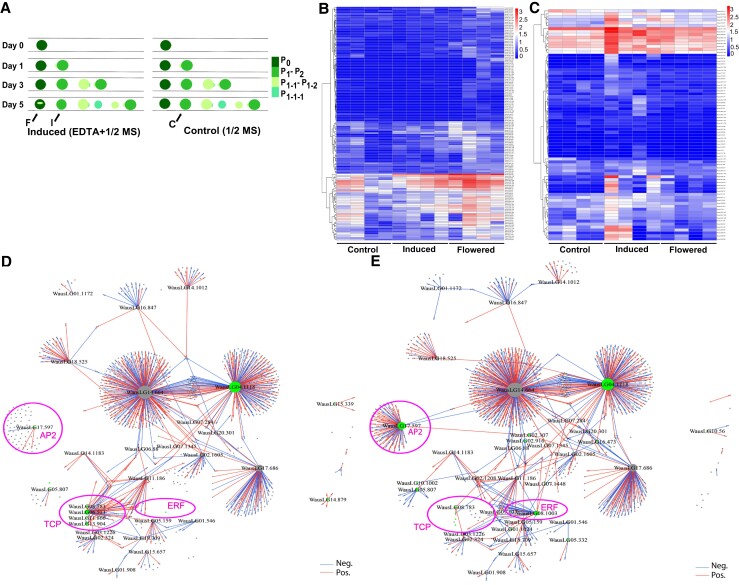
*W. australiana* floral induction and related transcriptome/TRNs. A) Diagram of the sampling design: F (flowered) samples were collected 5 days after culture under inductive conditions (left) from plantlets with a crack on the deck (shown as F with arrow). I (induced) samples were collected 5 days after culture under inductive conditions (left) from plantlets with no crack on the deck (shown as I with arrow). C (control) samples were collected 5 days after culture under noninductive conditions (right, control) from plantlets (shown as C with arrow) remaining in a vegetative state. B) Heat map representation of 147 differentially expressed genes that are up-regulated in flowered compared with induced (fold change ≥2 or ≤−2, *P* ≤ 0.05). C) Heat map representation of 78 differentially expressed genes that are down-regulated in flowered compared with induced (fold change ≥2 or ≤−2, *P* ≤ 0.05). D) TRN topological structure based on the comparison of the RNA-seq data sets from I and C samples. E) TRN topological structure based on the comparison of the RNA-seq data sets from F and C samples. Red edges for positive regulation, blue edges for negative regulation, green nodes for differentially present genes, and circled region for differentially present network in D) and E). Pink circles highlight the nodes exhibiting topological differences between the two TRNs.

Similar to *W. microscopia*, the transition from vegetative growth to induced flower morphogenesis is very fast and can take place in as few as 5 days in *W. australiana*, although not at a high frequency (Fig. S6). This quick transition and the simple reproductive structures (Fig. 1), combined with the PoC system to track the transition process of a single plantlet, prompted us to explore if the system could provide new opportunity to decipher the underlying regulatory mechanism. Accordingly, we collected three groups of individual plantlets for single-plantlet RNA-seq: (i) plantlets with floral organs 5 days after application of EDTA (ethylenediaminetetraacetic acid) treatment (to induce flowering), defined as F (flowered) samples; (ii) plantlets with no floral organs under the same inductive conditions (plantlet responses to flowering induction were not synchronized), defined as I (induced) samples; and (iii) plantlets grown for the same duration but not exposed to EDTA treatment, defined as C (control) samples (Fig. 5A). Using a candidate gene list of about 500 Arabidopsis and 100 rice flowering genes as queries, we identified about 200 homologous genes in *W. australiana* (Tables S14 and S16). Some of these genes exhibited differential expression between the C, I, and F groups, either up-regulated (Fig. 5B and Table S17) or down-regulated (Fig. 5C and Table S17). Among differentially expressed genes, we noticed that WausLG03.251 [homolog of *FLOWERING LOCUS T* (*FT*)] and WausLG11.346 [homolog of *FLOWERING PROMOTING FACTOR1* (*FPF1*)] are highly expressed in F samples (Tables S14 and S18), which was in agreement with the expression patterns of their Arabidopsis and rice homologs during the induction of flowering.

As an aquatic plant, the living conditions for *W. australiana* should be more stable than those of land plants because of the buffering capacity provided by water. Among the contracted gene families, we determined that miR156 is missing in *W. australiana* (Table S14). Although its target transcripts from *SQUAMOSA PROMOTER-BINDING-LIKE PROTEIN3* (*SPL3*) or the other family members *SPL4*, *SPL5*, *SPL9*, and *SPL15* were present in the *W. australiana* genome, the loss of miR156, as well as miR172 (Table S14), may explain the seemingly abrupt shift from vegetative growth to floral organ differentiation brought upon by the absence of the phase change observed in Arabidopsis and other plants normally mediated by the miR156-SPL module (23, 24).

Despite the loss of sepals and petals in the *W. australiana* flower, all MADS-box genes were present in the *W. australiana* genome and appeared to be expressed (Table S14). This observation thus raises a question about the genomic maintenance of MADS genes participating in sepal and petal identity determination in an organism lacking these organs over the course of evolution.

We also carried out a transcription regulatory network (TRN) analysis (25) with single-plantlet RNA-seq data to decipher the regulatory mechanisms behind floral organ initiation and differentiation. While the overall topological structures of TRNs between C-I and C-F pairs were quite similar, several network nodes distinguished the two TRNs (circled in Fig. 5D and E). While none of these distinct node genes were curated explicitly as known “flowering genes” (Table S19), the node genes exhibiting the most prominent differences (*P* = 1.65e^−8^, odds ratio = 60.90, hypergeometric distribution test) between the two treatments were those annotated as encoding APETALA2 (AP2)/ETHYLENE RESPONSE FACTOR (ERF) and TCP [TEOSINE BRANCHED1 (TB1), CYCLOIDEA (CYC), and PROLIFERATING CELL NUCLEAR ANTIGEN FACTOR1 (PCF1)] members (Fig. 5D and E and Table S19; note: although WausLG06.531 and WausLG08.783 are circled, they do not belong to the TCP family). TCPs were reported to be involved in floral organ formation (26). However, we determined that none of the *TCP* genes are differentially expressed in the C-F TRN, as expected based on the proposed function of Arabidopsis *TCP*s, although they were differentially expressed in the C-I TRN, that is from samples induced to flower but lacking clear floral organs (Fig. 5D and E). As AP2/ERF members have a wide range of functions in plants (26), the different TRN patterns for these two node genes raise interesting questions as to whether and how these genes function in *W. australiana* floral organ induction and differentiation.

### snRNA-seq, protein structure predictions, and gene transformation

A valuable model system should have a high-quality sequenced genome and should be amenable to genetic manipulation for the dissection of gene function. Here, we applied snRNA-seq and other techniques with *W. australiana*.

**Fig. 6.**
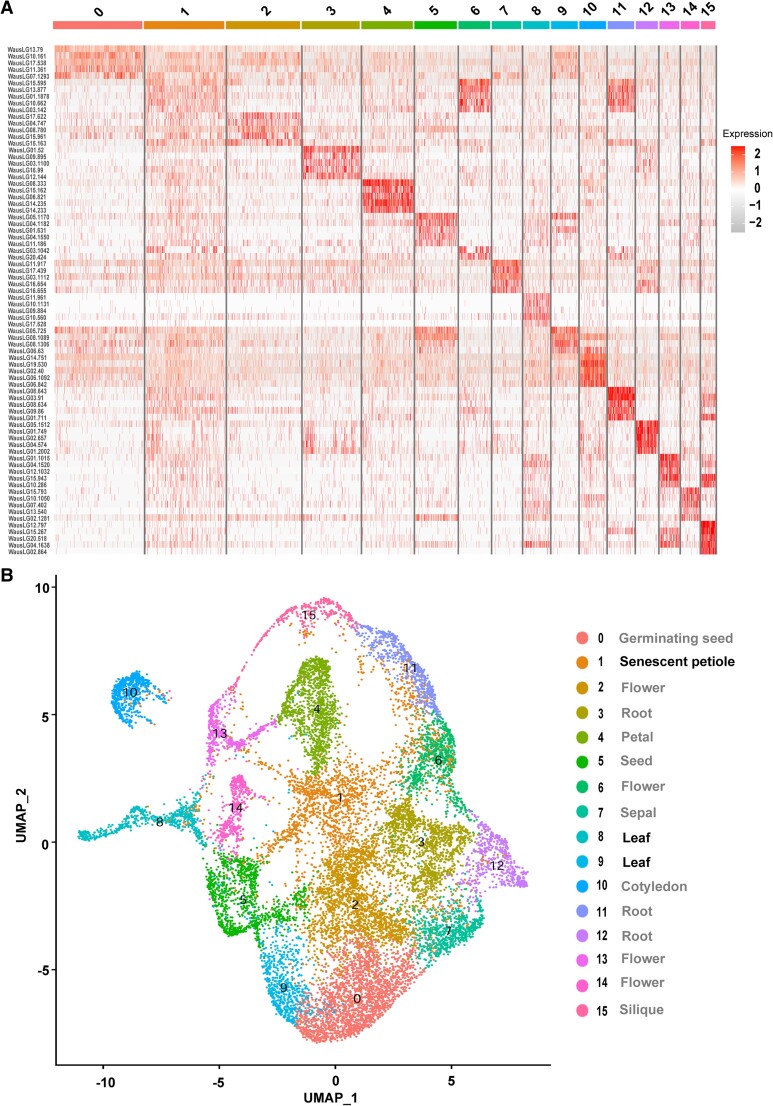
snRNA-seq reveals cell types absent in *W. australiana* plantlets. A) Heat map representation of differentially expressed genes across 15,983 cells clustered into 16 cell types. B) UMAP visualization of 15,983 cells into 16 clusters (for detailed information, see Table S16).

In a pilot experiment, we collected 2 g of plantlets cultured in flasks under regular growth conditions for nuclear isolation (27), followed by snRNA-seq (28). After data processing and quality control, we retained 15,983 nuclei and 14,812 genes for clustering, cell type annotation, and other analyses. Figure 6 illustrates the clustering pattern obtained by Uniform Manifold Approximation and Projection (UMAP); Table S20 provides the cell type annotation of each cluster. We were surprised to identify almost all cell types (Table S20; for full information, see Table S21), although the collected samples only consisted of boat-shaped leaves (with guard cells) and growth tips (Fig. 1). Notably, the results presented here are similar to those observed in snRNA-seq of freshwater sponge (*Spongilla lacustris*) (29). We will discuss this interesting phenomenon later.

We also tested the usefulness of the protein prediction software AlphaFold2 (30, 31), considering the importance of protein structures in biological structures and processes. Accordingly, based on our high-quality genome sequence and RNA-seq data, we selected 6,800 predicted nonhomologous protein sequences encoded by the *W. australiana* genome using MMseqs2 (32). With the non-Docker version of AlphaFold2, we predicted 6,798 structures and related information (https://www.wolffiapond.net). Only two predicted proteins failed to generate a structure due to video memory limitations. While the true protein structures remain to be experimentally examined, the high efficiency of protein structure prediction with AlphaFold2 to the *W. australiana* coding sequences was very encouraging.

Effective genetic transformation is an indispensable tool to manipulate the genome during functional investigations. In duckweed research, transgenic procedures have been reported in species other than *W. australiana* (14). We previously developed transgenic procedures for the stable transformation of *Wolffia globosa* and *S. polyrhiza* (L.) (33, 34). Here, we identified a set of modifications to the procedure to generate transgenic *W. australiana* plants (Fig. S8).

The PoC system is available for research teams interested in investigating fundamental questions in plant biology and plant development.

## Discussion

Here, we report that using *W. australiana* as a model system enables some fundamental issues in plant morphogenesis to be analyzed.

### Uncoupling the form and function of the growth tip in angiosperms

The growth tips of most angiosperms exhibit a tunica-corpus structure, while those of gymnosperms, pteridophytes, and bryophytes do not. Since the growth tips nevertheless carry out their functions as the centers of morphogenesis, the functional relevance of the tunica-corpus structure in angiosperms and how multicellular growth tips emerged remain elusive. Based on our observations in *W. australiana*, it is clear that the cell(s) located in the innermost regions of the cave behave as a growth tip, although they are not organized into a tunica-corpus structure (Fig. 1K). Under noninductive conditions, the growth tip continuously generates leaf primordia (Figs. 1 and S2). Under inductive conditions, the growth tip enlarges and protrudes further inward before forming one stamen and one gynoecium (Fig. 1N to Q). Based on these observations, we conclude that the tunica-corpus structure is dispensable for the proper function of the angiosperm growth tip, at least in the case of *W. australiana*.

The uncoupling of the form and function of the growth tip in angiosperms is not exclusive to *W. australiana*. Indeed, mutants in the Arabidopsis gene *WUSCHEL* (*WUS*) retain the ability to produce lateral organs from their growth tip for flowering, although the mutant lacks a typical tunica-corpus structure (35). One outstanding question worth pursuing is to investigate when and how the tunica-corpus structure evolved at the growth tip of angiosperms.

The emergence of axillary meristems has been explained by two rival hypotheses: de novo, the meristematic cells arise from differentiated somatic cells; and detached, the meristematic cells derives from preexisting meristematic cells (36). In agreement with a previous report (37), our observations on growth tip emergence during the differentiation of leaf primordia (Fig. 1I, J, L, and M) support the de novo hypothesis. In addition, compared with the in vitro regeneration of a SAM in tissue culture conditions (38), the relatively stable pattern of growth tip emergence in *W. australiana* makes it as an ideal experimental system to precisely investigate the spatiotemporal mechanism of the transition from partially differentiated somatic cells to meristematic cells.

### A unique opportunity for detailed analyses of the causal relationship between genome rewiring and morphogenetic simplification

The genome of *W. australiana* did not exhibit a dramatic size reduction relative to its close relatives *Colocasia esculenta* and *Zostera marina*, which produce multiple organ types in contrast to *W. australiana* (Fig. 2). Based on the genomic analyses presented here, we offer several clues that might explain this morphogenetic simplification: firstly, the *W. australiana* genome might have experienced a dramatic structural rewiring, as we detected 378 expanded gene families and 1,844 contracted gene families based on gene copy number, relative to its closest relatives (Fig. 2). Secondly, specific gene families showed a contraction in their constituent members. Members from the *AGL* family cluster into 11 groups in most plant species, whereas the *W. australiana* genome encoded AGL homologs belonging to nine groups (Fig. 2E). Similarly, the 13 Arabidopsis SCW–related *NAC* genes had a single homologous gene in *W. australiana*, which might be responsible for the nonvascular phenotype of this tiny plant (Fig. 4 and Table S15). Based on recent findings of the dynamic features of chromatin structures, it is reasonable to hypothesize that the relative stable aquatic conditions experienced by *W. australiana* may have contributed to genome rewiring, including gene and/or gene family contraction.

Other regulatory mechanisms may have also participated in the morphogenetic simplification of *W. australiana*. For example, the simple absence of a gene or gene family cannot explain all cases of morphological innovations, as we identified almost all known genes required for Arabidopsis root development in the *W. australiana* genome (Table S14). More curiously, our pilot snRNA-seq experiment revealed the expression of genes that are typically markers for tissues or organs missing in *W. australiana* plantlets. Notably, the genome of freshwater sponges was recently shown to harbor genes involved in nerve cell development, although this organism lacks this cell type (29). It is possible that the expression of certain genes may occur prior to the emergence of a given morphogenetic event. Before the genes were coopted for specific morphogenetic events, they may have carried out other functions. Our high-quality genome enables to explore the relationships between gene annotation and morphogenetic events.

### Retracing cell fate transition from vegetative to reproductive growth in an individual growth tip

Flowering is one of the morphogenetic processes in angiosperms, which has attracted the most interest in plant research. While significant progress has been made over the past century, a thorough dissection of the underlying regulatory mechanisms has been hindered by two limitations: (i) the transition from vegetative to reproductive growth is a long process consisting of sequential changes from photosynthetic leaves to peripheral organs, with the added interference of complex branching in an asynchronous system; and (ii) the transition from vegetative to reproductive growth integrates multiple internal and external environmental changes within each growth tip, which may respond quantitatively and qualitatively differently (2). A simpler experimental system would be especially helpful here.

With the PoC system described here, we easily traced the entire transition from vegetative to reproductive fate of an individual growth tip. Furthermore, we were able to trace how the growth tip differentiates into two primordia upon flowering induction (Fig. 1N to P), which will further differentiate into the stamen and gynoecium, all within a time frame of a few days (Fig. 1Q, F, and G). In addition, we identified simple but efficient inductive conditions to trigger the transition. Therefore, with the assistance of cutting-edge single-cell techniques, this PoC system should provide a unique opportunity to decipher the regulatory mechanisms that drive fate transition with a much lower signal-to-noise ratio.

The simplicity of the *W. australiana* floral structure and accessibility may offer a novel approach, to the regulatory mechanism of flower development and evolutionary origin. Based on our observations (Fig. 1N to Q), the stamen and gynoecium are aligned at the ends of separated branches. If the stamen and gynoecium in *W. australiana* are considered as elaborated heterosporangia (39, 40), the origin of the flower may be seen as a result of two separate evolutionary innovations: the transition from homosporangium to heterosporangia and the combination of individually initiated fertile telomes, each with microsporangium and megasporangium, together as a morphological unit recognized as a “flower.” *W. australiana* may thus be amenable to the exploration of how the two primordia emerge upon induction and how they differentiate into a stamen or gynoecium. Such dissection should provide a substantial basis for the investigation of heterosporangia differentiation in more species from Pteridophytes to Spermatophytes.

Although we have yet to explore fertilization and seed formation in the *W. australiana* life cycle, we did precisely visualize and trace most of the morphogenetic events required for sporophyte development at the cellular level. We have illustrated the possible causal relationship between morphological traits and genome variation for root and vascular differentiation. Other interesting morphogenetic events will be open to investigation, such as the programmed cell death that results in the formation of the crack on the “deck” of the boat-like plantlets; how guard cells differentiate on the “deck” but not on the “hull”; and how the asymmetric growth of the leaf primordium is guided. The PoC system established here can offer a unique opportunity for deciphering the regulatory mechanisms of core processes of plant development and other interesting morphogenetic events during the sporophyte stage.

## Materials and methods

### Biological materials

*W. australiana* wa7733 was from Prof. Hongwei Hou Lab at the National Aquatic Biological Resource Center, Institute of Hydrobiology, Chinese Academy of Sciences. *W. australiana* plants used for genome sequencing and transcriptome sequencing were cultured in half MS (Solarbio) solid medium (1% sucrose, pH 5.5) under long-day (16-h light/8-h dark) conditions at 26°C. The fresh plants cultured for 10–15 days were used for the extraction of genomic DNA for genome sequencing and total RNA for transcriptome sequencing from plants in a population under cultured conditions (Table S1).

The *Wolffia* plants were grown in liquid half MS medium (1% sucrose, pH 7 and antibiotics free), under short-day (8-h light/16-h dark) conditions at 26°C on the plates for 1–2 weeks. Plants were transferred to half MS medium (control, C) or half MS + EDTA (Sigma) medium (induced but not flowered, I and flowered, F) in chip for 5 days before RNA extraction for single-plant RNA-seq. Each group (C, I, and F) included four single *Wolffia* plants as four biological replicates.

The *Wolffia* plants were grown in half MS medium, under short day at 26°C on the plates. The fresh weight of *Wolffia* was 2 g for one sequencing for snRNA-seq.

### Floral organ staining

The flowered *W. australiana* was (i) stained in Direct Red 23 (1/100) over 8 h; (ii) transferred to transparent solution (6-g trichloroacetaldehyde in 0.6-mL glycerol and 2.2-mL H_2_O) until clear; and (iii) washed in H_2_O three times with gentle shaking.

### Photomicrograph conditions

We photographed and shot time-lapse recording with a microscope digital camera (Olympus, DP27) or incubator's camera. The shooting time was based on the experimental design.

### PoC device design

We developed a light culture method to study *Wolffia* using a new PoC system. The chip was made of PDSM (mixed Momentive and RTV615). PDSM (the ratio of Momentive:RTV615 is 10:1) was poured into mold. The mold was placed into a 70°C oven for 4 h and cut into pieces before use. The mold was printed by a 3D printer (Fig. S5), with 1-mm channel width, 10-mm depth, and 35-mm length. A single *W. australiana* was planted into the channel of chip in clean bench and submerged in medium. The set was connected to the pump (MesoBioSystem) for medium cycling (the pump rate could be set 0–8 times/s). All the solutions and components were sterilized before use because there were no antibiotics. The set and pump were transferred into incubator without destabilization. This custom-made incubator includes five systems: (i) light, (ii) thermostat, (iii) camera and microscope, (iv) control and display, and (v) cabin (Fig. S5). The blueprint for the incubator was shown in Fig. S5. The culture conditions could be set on the touch-screen controller or the external computer.

### Cryo-scanning electron microscopy

Cryo-SEM was used to study the *Wolffia* morphology. The equipment included the Helios NanoLab G3 UC scanning electron microscope (Thermo Fisher Scientific) and the PP3010T workstation (Quorum Technologies), which had a cryo-preparation chamber connected directly to the microscope. The *Wolffia* were frozen in subcooled liquid nitrogen (−210°C) and transferred in vacuum to the cold stage of the chamber, where sublimation (−90°C, 5 min) and sputter coating (10 mA, 60 s) with platinum were conducted. Finally, the samples were transferred to another cold stage in the scanning electron microscope and imaged. The image was recorded using the electron beam at 5 kV and 0.2 nA with a working distance of 10 mm. The resolution of the final data was 3,072 × 2,048.

### Sample preparation for micro-CT and TEM

*Wolffia* plantlets were fixed with 2% (w/v) glutaraldehyde (Sigma-Aldrich, G7651) and 2% (w/v) paraformaldehyde (Sigma-Aldrich, P6148), 0.1 m phosphate buffer (81 mm Na_2_HPO_4_ (Sigma-Aldrich 71649), and 19 mm NaH_2_PO_4_ (Sigma-Aldrich 71507), pH 7.4, for 2 h at room temperature, and then held overnight at 4°C. The next day, the samples were washed with 0.1 m phosphate buffer for three times and postfixed in 2% (w/v) OsO_4_ (Ted Pella, 18459) and 1.5% (w/v) potassium ferrocyanide (Sigma-Aldrich, P3289) for 2 h at 4°C. Following five washes with phosphate buffer and distilled H_2_O, samples were put into 1% thiocarbohydrazide (Sigma-Aldrich, 223220) and followed by another postfixation in 1% (w/v) OsO_4_, and three times of washing with distilled H_2_O were also included after each step. At last, samples were dehydrated through a graded alcohol series and embedded in Spurr's resin (SPI Supplies, 02680-AB).

Resin blocks were used for micro-CT, and the pictures were taken by Xradia Context (Zeiss) or SkyScan 1272 (Bruker). Next, selected resin blocks were cut into 70-nm sections using an ultramicrotome (Leica Microsystem, UC7) referring to the micro-CT data for transmission electron microscopy (TEM) (JEOL, JEM-1400).

### Genome sequencing

Genomic DNA was extracted from a *W. australiana* sample collected from a population using SDS method to construct ultralong DNA libraries using above 10 *μ*g DNA (with size selected ≥50 kb) and the SQK-LSK109 sequencing preparation kit (Nanopore, Ligation Sequencing Kit). The samples were sequenced on the Promethion (Oxford Nanopore Technologies, UK) at the Genome Center of Grandomics (Wuhan, China). The same genomic DNA was used to produce the short-read Illumina sequencing library by performing g-TUBE fragmentation, repair, adaptor connection, PCR amplification, and recycling ∼400-bp sequence using ∼2-*μ*g DNA and then sequenced on the Illumina NovaSeq 6000 platform at Grandomics (Wuhan, China). As for the Hi-C data, freshly prepared samples were chopped into 2-cm pieces and vacuum infiltrated in nuclei isolation buffer supplemented with 2% formaldehyde. Cross-linking was stopped by adding glycine and additional vacuum infiltration. Fixed tissue was then grounded to powder before resuspending in nuclei isolation buffer to obtain a suspension of nuclei. The purified nuclei were digested with 100 units of DpnII and labeled by incubating with biotin-14-dCTP. Biotin-14-dCTP from nonligated DNA ends was removed because of the exonuclease activity of T4 DNA polymerase. The ligated DNA was sheared into 400-bp fragments and then was blunt end-repaired and A-tailed, followed by purification through biotin–streptavidin-mediated pull down. Next, for Bionano physical mapping, DNA extracted from population plants of *W. australiana* was subject to library preparation with Bionano Prep Plant DNA Isolation Kit (Bionano Genomics, Cat# 80003) following manufacturer recommended protocols. Optical scanning was provided by Bionano Genomics (https://bionanogenomics.com), with Bionano Prep DLS Labeling DNA Kit (Bionano Genomics, Cat# 80005). Labeled DNA samples were loaded and run on the Saphyr system (Bionano Genomics) in Grandomics. Finally, the Hi-C libraries were quantified and sequenced using the Illumina NovaSeq 6000 platform. To obtain RNA-seq, total RNA was extracted from different samples in TRIzol reagent (Invitrogen, Cat# 15596018)/CTAB-LiCl method (Plant) on dry ice and processed following the manufacturer's protocol. Sequencing libraries were generated using TruSeq RNA Library Preparation Kit (Illumina, USA) following standard protocol. Briefly, ∼1-*μ*g RNA per sample was used and enriched from total RNA using oligo(dT)-attached magnetic beads. The first-strand cDNA was synthesized with random primer and M-MLV Reverse Transcriptase, and the second-strand cDNA synthesis was followed by using DNA Polymerase I and RNase H. The synthesized cDNA was end-repaired, A-tailed, and ligated to the sequencing adapters according to library construction protocol. The cDNA fragments were selected by AMPure XP beads (Beckman Coulter, USA) to an average size of 150–200 bp and amplified by PCR with Phusion High-Fidelity DNA polymerase, Universal PCR primers, and Index Primer. At last, PCR products were purified with AMPure XP Beads (Beckman Coulter, USA), and library quality was assessed on the Agilent Bioanalyzer 2100 system. After that, the library was sequenced on the Illumina NovaSeq 6000 platform.

### Genome size estimation

The genome size of *W. australiana* was estimated using the K-mer method based on the Illumina NovaSeq 6000 next-generation sequencing (NGS) data. Firstly, the quality-filtered paired-end data were filtered by Fastp v0.19.4 with specific parameters (-f 3 -t 2 -F 3 -T 2 -n 0) and then sequences of bacteria, mitochondria, and chloroplast were removed from the quality-filtered reads to produce the complete genome data by aligning against a bacteria and organelle database with BLASTN v2.9 with the parameter “E-value 1e^−5^”. Secondly, the K-mer size was selected to be 17 bp, and total number and average depth of K-mer sequences were calculated from the complete genome data reads (Table S2). Finally, the genome size was obtained by calculation based on the formula: genome size = total K-mer number/average K-mer depth.

### Chromosome-level genome assembly and assessment

NextDenovo v2.0-beta.1 (https://github.com/Nextomics/NextDenovo/) was used with the filtered ultralong sequences to produce the initial genome sequence (G1) with these parameters “reads_cutoff = 1k seed_cutoff = 50k” and “-n 1966 -q 0 -i 0.41 -s 0.18 -n 2 -r 0.14 -m 7.88 -c 80 -z 12”. Then, the ultralong passed reads and the genome quality-filtered paired-end reads were applied to polish the G1 genome sequences by NextPolish v1.0.5 with the parameters (task = 55512121212) to produce polished genome (G2). To eliminate contamination sequences of the genome which may result in potential problems in the downstream analysis, the G2 contigs were aligned to the Nucleotide Sequence Database (NT) by using BLASTN v2.9 with the parameter “E-value 1e-5”, and the sequence alignment results were classified based on species taxonomy. The contig was aligned to bacteria genome and was classified to contamination sequences and filtered out from G2 to form the decontaminated genome (G3).

To evaluate the accuracy of the G3, Illumina genomic paired-end reads were mapped to the genome contig sequences by the “mem” submodule of BWA. The mapping identity and genome coverage of the genome assembly were calculated based on the mapping result obtained by SAMtools v1.4 with default parameters. Homozygous single-base variations were subsequently detected by using BCFtools v1.8.0 with default parameters. Furthermore, Illumina RNA-seq reads were mapped to the genome sequence by using HISAT2 v2.1 with default parameters, and the mapping rate of RNA-seq reads was calculated by SAMtools. The completeness of conserved genes and eukaryote core genes assembly were evaluated by using BUSCO v3.1.0 with the “embryophyta_odb10” data set and CEGMA v2 with default parameters, respectively. Meanwhile, 10-kb bins were formed from G2 and were used to build the distribution of GC-Depth.

To obtain accurate genome, the Bionano physical mapping was used to map the G3 by Bionano Solve data analysis software to form the G4 version of genome. To anchor the G4 sequences to 20 chromosomes, the original Hi-C paired-end reads generated by the Illumina platform were aligned to the final contig sequences by Bowtie2 v2.3.2 with parameters “-end-to-end --very-sensitive -L 30”. The contigs were clustered and ordered by using LACHESIS (41) with parameters “CLUSTER MIN RE SITES = 100; CLUSTER MAX LINK DENSITY = 2.5; CLUSTER NONINFORMATIVE RATIO = 1.4; ORDER MIN N RES IN TRUNK = 60; ORDER MIN N RES IN SHREDS = 60”. Finally, the genome contigs were scaffolded into chromosome sequences to get the chromosome-level genome (G5) sequences (Table S3) and Hi-C interaction heat map was obtained based on the contig interaction results.

### Genome repeat and gene annotation

Simple sequence repeats (SSRs) and TR sequences were firstly identified from the chromosome-level genome by GMATA v2.2 with default parameters and Tandem Repeats Finder v4.07b with parameters “2 7 7 80 10 50 500 -f -d -h -r”, respectively. Sequence identified as TR sequences were “soft masked” as lowercase letters in the genome. Secondly, LTR retrotransposons were identified from the TR-masked genome assembly using LTR_finder v1.07 and LTR_harverst v1.5.10 with default parameters separately; a LTR-repeat library was constructed by LTR_retriver v1.8.0 with default parameters based on the results above. MITEs were subsequently identified using MITE-Hunter v11–2011 (42) with parameter “-n 20 -P 0.2 -c 3”. The identified LTRs and MITEs were combined to mask the *W. australiana* genome and novel TEs were identified by RepeatModeler v1.0.11 (https://github.com/Dfam-consortium/RepeatModeler) to construct the TE library. Finally, LTRs library, TEs library and Repbase were combined into one library file as the input for RepeatMasker with parameters “nolow -no_is -gff -norna -engine abblast -lib lib” to search repetitive sequences throughout the genome.

Protein-coding genes were predicted from the genome using the EVidenceModeler (EVM) pipeline v1.1.1 which integrates gene models originated from three sources of predictions: de novo, protein homology and transcriptome prediction. Homologous proteins of 15 plant species (Tables S11 and S12) across the Viridiplantae were used by GeMoMa v1.6.1 (43) with default parameters. Illumina RNA-seq reads were applied to predict genes by using HISAT2 (default parameters), StringTie v1.3.3d (default parameters), and PASA v2.3.3 (-C -R -g -T -u -t -f --ALIGNERS gmap). Predicted genes based on the transcripts were compared with SWISS-PROT Database by BLASTP v2.9 with the minimum identity requirement of 95%, and then the top 3,000 hits were selected as the training sets to train AUGUSTUS v3.3.1 with default parameters. De novo prediction evidence, protein homology evidence, and transcript evidence were combined and the gene models of the *W. australiana* genome were predicted by EVM with parameters “--segmentSize 1000000 --overlapSize 100000”. The obtained EVM genes were aligned against the TransposonPSI (https://transposonpsi.sourceforge.net/) database to exclude predicted genes containing TE-related domains. Genes with length that could not be divided by 3 were also excluded based on the principle of codon translation. The protein sequences were subsequently mapped to various functional annotation databases by BLASTP v2.9 with parameters “-evalue 1e-5, -max_target_seqs 1”, including nonredundant (NR) protein sequence databases, Kyoto Encyclopedia of Gene and Genomes (KEGG) database, SwissProt database, and Eukaryotic orthgene Groups of protein (KOG) database. The Gene Ontology (GO) analysis was performed by InterProScan v5.32–71.0 (44) with default parameters. To further assess the quality and completeness of the predicted gene model, protein sequences were aligned to the “embryophyta_odb10” data set of BUSCO v3.1.0 to evaluate the completeness of conserved gene set.

### Genome phylogenetic analysis

*W. australiana* and 14 other species (Table S12) were included for phylogenic evolutionary analysis. The OrthoMCL pipeline v2.0.9 was used to identify gene families between genomes of these species, and the protein sequences of the longest transcript of each gene among these species were mutually aligned using BLASTP with parameters “-evalue 1e-5” to obtain the sequence similarity information. Orthologous genes and paralogous genes were subsequently classified. Orthologous single-copy genes were used as the seed sequences to perform the phylogenetic analysis. The single-copy gene sequences were aligned by MAFFT v7.313, and then the above result was trimmed by Gblocks v0.91b with parameters “-t = p -b5 = h” to construct a phylogenetic tree based on the GTRGAMMA model and 1,000 bootstrap replicates by RAxML v8.2.10 (-m PROTGAMMAAUTO -p 12345 -T 8 -f b). Divergence time obtained by TimeTree was set as calibration points (Table S13) to divergence time estimation among 15 species with RelTime by default parameters. Gene family contraction and expansion analysis was performed by CAFE v4.2.1 with parameters “-p 0.05 -t 10 -r 10000”, which applies a birth and death rate to model gene family size over a phylogeny based on orthologous groups from OrthoMCL and the ultrametric tree obtained from RelTime. Expanded or contracted gene families with viterbi *P* ≤ 0.05 and family-wise *P* ≤ 0.01 were defined as significant compared with the last common ancestor. Both GO and KEGG enrichment analyses of significant gene families were performed by clusterProfiler Package (45) with parameters “pAdjustMethod = ‘BH’, pvalueCutoff = 1, qvalueCutoff = 1”.

### RNA extraction and library preparation for single-plant RNA-seq

Single-plant *Wolffia* RNA was extracted using the protocol of RNeasy plant mini kit (Qiagen, cat# 74903). RNA concentration was measured using a Qubit RNA assay kit (Thermo Fisher, Ref# Q32855A) in a Qubit 2.0 fluorometer (Life Technologies, Ref# REQ32866). Total RNA of 100 ng per sample was used as input material for RNA sample preparations.

Libraries were generated using a NEBNext Ultra RNA library prep kit Illumina (New England Biolabs, Ipswich, MA, USA, Cat# E7530), and index codes were added to attribute sequences to each sample. The mRNA was purified from total RNA using poly-T oligo-attached magnetic beads (New England Biolabs, Ipswich, MA, USA, Cat# E7490). First-strand cDNAs were synthesized by the NEBNext RNA First Strand Synthesis Module (New England Biolabs, Ipswich, MA, USA, Cat# E7525L). Second-strand synthesis was performed by the NEBNext Ultra II Non-Directional RNA Second Strand Synthesis Module (New England Biolabs, Ipswich, MA, USA, Cat# E6111L). Library was prepared using KAPA Hyper Prep Kit (KAPA Biosystems, Wilmington, MA, USA, Cat# KK8504). Products were purified with AMPure XP system (Beckman Coulter, Cat# A63882), and library quality was assessed using the Agilent Bioanalyzer 2100 system (Agilent Technologies, Ref# G2939BA). The library was sequenced on the Illumina HiSeq 4000 platform.

### Analysis of genes related to morphological processes

The *A. thaliana* root and flower genes (Table S14) were used as the seed sequences to align against the gene set of *W. australiana*. Meanwhile, we used the same method to the gene set of *Oryza sativa* (Table S16). Then, these genes were classified to different gene families based on the previous research of *A. thaliana*. Next, each of gene family was used to construct gene tree by MEGAX (sequences aligned by MUSCLE and tree constructed by maximum likelihood method).

### DEG analysis

Different data sets of transcriptome (control, C; induced, I; and flowered, F) of the *W. australiana* were obtained (Table S1). Based on the different transcriptome data sets, read counts of genes in each sample obtained from hisat2 and StringTie with default parameters, differential expression genes (DEGs), and among different sample comparisons were identified by DESeq2 Package (46). Genes with a false discovery rate (FDR) ≤0.05 and fold change ≥2 were considered to be DEGs in each comparison (Table S17). Both GO and KEGG enrichment analyses of DEGs in each group were performed using clusterProfiler Package with parameters “pAdjustMethod = ‘BH’, pvalueCutoff = 1, qvalueCutoff = 1”.

### Chromosome FISH analysis

Chromosome preparation was conducted according to a previous study (47). The entire plants were harvested and pretreated in 0.002 m 8-hydroxyquinoline at 23°C for 2 h and then fixed with Cannoy Solution (methanol: acetic acid = 3:1) at 25°C overnight. After washed with distilled water, the plants were digested using the enzyme mixture of 2% cellulase and 1% pectinase at 37°C for 90 min. After that, they were quickly rinsed in distilled water and fixed with the same fixation solution. The samples were spread on precooled slides and dried by passing through the flame of a Bunsen burner three or four times. Chromosomes were counterstained with 4′,6-diamidino-2-phenylindole (DAPI) in an antifade solution (Vector Laboratories, Burlingame, CA, USA). The FISH assay was conducted using 45S recombinant DNA (rDNA) as a probe labeled with digoxigenin and detected by antidigoxigenin rhodamine (48). Images were captured under a Zeiss A2 fluorescence microscope with a microcharge-coupled camera (Zeiss, Germany).

### Microscopy

The *W. australiana* and *S. polyrhiza* plantlets were macerated with 10% peracetic acid at 80°C for 30 min. After extensive washing, the plantlets were squashed and stained with cell wall dye Direct Red 23 (0.01% v/v) and photographed using a fluorescence microscope (Imager D2, Zeiss).

To test the function of the only SCW–related VND homolog *WausLG14.977* in vessel differentiation, the full-length coding sequence of *WausLG14.977* was cloned and inserted into a binary vector pCAMBIA1300 between *CaMV 35S* promoter and *NOS* terminator. The resulting construct was transfected into *Agrobacterium tumefaciens* strain EHA105 and infiltrated into 1-month-old tobacco leaves. Autofluorescence signals of the induced vessel wall were recorded with 405-nm excitation using a laser scanning confocal microscope (LSM 980, Zeiss).

#### Cell wall composition analysis

The *W. australiana* plantlets were collected, freeze-dried, and subjected for cell wall composition determination as described previously (49). In brief, the alcohol insoluble residues were prepared by ball milling and successive extraction and then incubated with α-amylase (Sigma) at 97°C for 35 min and 60°C for 1 h to remove starch. The destarched alcohol insoluble residues were mildly hydrolyzed using 2 m trifluoroacetic acid. The supernatants were treated with sodium borohydride solution to reduce alditols and then with acetic anhydride to acetylate. The resulting alditol acetates were extracted with ethyl acetate and quantified using gas chromatography (7890, Agilent)-coupled mass spectrometry (5977, Agilent). The pellets remained were treated in Updegraff reagent (acetic acid:nitric acid:water, 8:1:2, v/v) at 100°C for 30 min and further hydrolyzed with 72% (v/v) sulfuric acid. Cellulose content was measured via anthrone assays. Five biological replicates were included in the examinations.

### Phylogenetic analysis

A neighbor-joining phylogenetic tree of SCW–related NAC homologs in *W. australiana*, *P. patens*, *Selaginella moellendorffii*, *Amborella trichopoda*, *O. sativa*, and *A. thaliana* was built using MEGA6 software (50) with 1,000 bootstrap replicates. The sequences were obtained from the online server PLAZA (https://bioinformatics.psb.ugent.be/plaza/), except that the sequences of *W. australiana* homologs were obtained from this study. Sequence alignment of WausLG14.977 with the NAC homologs, AtSND1, OsSNAC1 (3ULX), AtANAC (1UT7), and AtANAC019 (3SWM), was conducted using ClustalW (https://www.clustal.org) and ENDscript/ESPript (https://endscript.ibcp.fr).

The 3D protein structures of WausLG14.977 and AtSND1 (At1g32770) were predicted by using the RoseTTAFold server (https://robetta.bakerlab.org) according to the instructions (51). The predicted model 1 was displayed and applied for structure comparison using UCSF Chimera (https://www.rbvi.ucsf.edu/chimera). The protein structures of NAC homologs SNAC1 (3ULX), ANAC (1UT7), and ANAC019 (3SWM) were downloaded from RCSB PDB database (https://www.rcsb.org/). *Z*-scores of WausLG14.977 protein structures to that of Arabidopsis SND1 and rice SNAC1 were determined using the Dali server (https://ekhidna2.biocenter.helsinki.fi/dali).

### TRN analysis

To build the regulatory network, we first called putative transcription factors (TFs) as well as corresponding binding motifs in *W. australiana* genome based on the established pipelines (25) then scanned the promoter regions of all predicted *W. australiana* genes (TSS −500 bp to +100 bp) with the identified motifs systematically (by FIMO, cutoff 1e^−5^) to connect the TFs and their potential targets. The inferred network was further refined via transcriptomic data with the following criteria: (i) the TF-target pair should be coexpressed (Spearman correlation >0.9); (ii) at least one gene in the TF-target pair should be differentially expressed between control samples and flowered samples or induced but not flowered samples, and (iii) at least one gene in the TF-target pair should be the floral organ initiation or differentiation related genes (Table S19).

### Protein structure prediction and classification

The predicted mRNAs were firstly translated into protein sequences. MMseqs2 (32) program suite was employed for further protein sequence analysis; cluster submodule was then used for clustering to reduce redundancy of sequence space, with *e*-value threshold set to 1e^−5^. Representative sequences of clusters were compared with those available in structure database (i.e. PDB (52, 53) and AlphaFold Protein Structure Database (31) using search submodule, with *e*-value threshold set to 1e^−5^ as well). As a result, 6,800 nonhomologous protein sequences were left for further structural prediction.

Non-Docker version AlphaFold2 (30) was deployed for speed and scalability. Features (i.e. multiple sequence alignments) needed as input for further prediction were firstly generated on a distributed cluster of machines without GPUs. Further structural prediction by neural network and refinement using molecular dynamics were both conducted on machines with graphics cards. Each task was provided with one graphics card to speed up computation. Finally, we obtained 6,798 predicted structures and their relative information, while the prediction for the other two failed due to video memory limitation.

To identify the superfamily of these predicted structures, we used DaliLite.v5 (54) (i.e. a standalone program for protein structural alignment using Dali method) to compare these with representative structures of superfamilies provided by SCOPE (55, 56). The all-against-all structural comparisons were performed with default parameters. The hits with the highest *Z*-score were considered as the best ones, and thus the superfamilies of query predicted proteins were considered as same as those of best hits.

### Single-nucleus isolation, single-nucleus library construction and sequencing, raw data processing, and generation of gene expression matrix

Fresh samples of *W. australiana* were used for single-nucleus isolation as described previously (27, 57) with slight modifications. The single-nucleus library construction and sequencing were conducted as previously described (58). The DNBelab C Series Single-Cell Library Prep Set (MGI, Cat# 1000021082) was utilized as in Liu et al. (28).

Raw reads were demultiplexed and mapped to the Waus reference genome by PISA (Version 1.1.0). The pipelines include align reads and generate gene-cell matrices. STAR was used with default parameters for mapping and transcript quantifications. The gene-cell matrices generated by R package Seurat were used for subsequent analysis.

### Cell clustering, cell type identification, quality control, and cell type annotation for snRNA-seq

Downstream analyses were mainly performed with Seurat. The gene-cell matrices of 42,667 cells were loaded into the Seurat package implemented in R (Version 3.6.3). The analysis was performed as previously described (58).

To filter out the low-quality cells, the following quality control criteria were followed: one gene is expressed in ≥3 cells, and one single cell expresses ≥200 genes. We filtered the cells with gene numbers >3,000 or <200, and unique gene counts <500. After applying these quality control criteria, 15,983 cells and 14,812 genes were kept for subsequent analysis.

The cell type of each cell cluster was manually defined by the high-expressed genes in each cell cluster, based on these cluster-enriched genes’ correspondence to Arabidopsis genes. The Arabidopsis genes’ expression information was retrieved from BAR eFP Browser (59) from TAIR (The Arabidopsis Information Resource) (https://www.arabidopsis.org/index.jsp).

### Gene transformation of *W. auatraliana*

The *A. tumefaciens* strain LBA4404 harboring the plasmids of synthetic reporters—TCS::GUS/pKGWFS7.0 which have been used to report cytokinin output was used in our study (34). Approximately 0.5-g fresh *Wolffia* and 1-g sterilized glass beads (1 mm) were placed in 2-mL sterilized Eppendorf microcentrifuge tube with 500-*μ*L *Agrobacterium* suspension, 1-*μ*L silwet L-77, and 100 *μ*m acetosyringone (AS). The tubes were first subjected to ultrasound at a frequency of 40 kHz for 1 min at 28°C. After sonication treatment, a vacuum of ∼0.7 kg/cm^2^ was applied for 10 min. Then, the tubes were shaken around 150 rpm for 15 min at 28°C. Finally, plants were transferred onto filter papers wetted with liquid half SH medium adding 1% sucrose (w/v) and 100 *μ*m AS at pH 5.2 in the dark for 5 days at 25°C.

After 5 days of cocultivation, the infected *Wolffia* plants were transferred to selection medium: solid half SH medium supplemented with 300 mg/L cefotaxime, 20 mg/L G418 (Geneticin), and 1% (w/v) sucrose under a 16-h/8-h photoperiod of ∼85 *μ*mol m^−2^ s^−1^ of white light at least for 4 weeks. Then, the survived plants were transferred to induction medium: solid half SH medium adding 150 mg/L cefotaxime, 20 mg/L G418 (Geneticin), and 1% (w/v) sucrose. The resistant plants were identified by the β-glucuronidase (GUS) assay. The transformation efficiency was calculated as percentage of GUS-positive plants in the total number of explants. The highest transformation efficiency in current study was ∼46%.

Note: all materials are listed in Supplementary Table S22.

### Statistical analysis

All details of the statistics applied are provided alongside in the materials and methods, figures and tables, and the corresponding legends.

## Supplementary Material

pgad141_Supplementary_Data

## Data Availability

The genome sequence of *W. australiana* wa7733 has been deposited at NCBI Genome under the accession CP092600-CP092619 for 20 chromosomes. Raw genome and transcriptome sequencing reads have been deposited into the NCBI sequence read archive (SRA) under the BioProject PRJNA808652 (for Nanopore), PRJNA808655 (for Illumina genome), PRJNA808685 (for Hi-C), PRJNA808734 (for BioNano), PRJNA808736 (for RNA-seq for genome), PRJNA808739 (for single-plant RNA-seq), and PRJNA809022 (for single-nucleus RNA-seq). BioNano data have also been deposited into NCBI Supplementary Files under the accession SUPPF_0000004267. Genome sequence and single-plant and single-nucleus RNA-seq are also available at the *W. australiana* wa7733 genome database: https://wolffiapond.net.
