# Supplementary material for: Plant-on-chip: Core morphogenesis processes in the tiny plant Wolffia australiana
Source: PNAS Nexus. 2023 Apr 19;2(5):pgad141. doi: 10.1093/pnasnexus/pgad141 (PMC10169700; doi:10.1093/pnasnexus/pgad141)
Supplement: pgad141_Supplementary_Data [file pgad141_supplementary_data.zip › supp SI 042923 proof REF.docx]

Supplementary Material for

**Plant-on-Chip: core morphogenesis processes in the tiny plant** ***Wolffia australiana***

Feng Li^a,b,c,d,†^, Jing-Jing Yang^e,†^, Zong-Yi Sun^f,†^, Lei Wang^g,†^, Le-Yao Qi^a^, Sina A^a^, Yi-Qun Liu^d^, Hong-Mei Zhang^d^, Lei-Fan Dang^d^, Shu-Jing Wang^b^, Chun-Xiong Luo^b^, Wei-Feng Nian^a^, Seth O’Conner^g^, Long-Zhen Ju^f^, Wei-Peng Quan^f^, Xiao-Kang Li^f^, Chao Wang^f^, De-Peng Wang^f^, Han-Li You^h^, Zhu-Kuan Cheng^h^, Jia Yan^d^, Fu-Chou Tang^d^ , De-Chang Yang^c,d,i,j^, Chu-Wei Xia^c,d,j,j^, Ge Gao^c,d,i,j^, Yan Wang^h^, Bao-Cai Zhang^h^, Yi-Hua Zhou^h^, Xing Guo^k^, Sun-Huan Xiang^k^, Huan Liu^k^, Tian-Bo Peng^c,d^, Xiao-Dong Su^c,d^, Yong Chen^l^, Qi Ouyang^b,m^, Dong-Hui Wang^c,d^, Da-Ming Zhang^n^, Zhi-Hong Xu^c,d^, Hong-Wei Hou^e,*^, Shu-Nong Bai^b,c,d,*^ and Ling Li^g,*^

^*^To whom correspondence may be addressed. Email: [houhw@ihb.ac.cn](mailto:houhw@ihb.ac.cn) (H.-W.H.) or [shunongb@pku.edu.cn](mailto:shunongb@pku.edu.cn) (S.-N.B.) or liling@biology.msstate.edu (L.L.)

^†^These authors are joint first authors and contributed equally to this work.

This file includes:

Figs. S1 to S8

Table S22

Other Supplementary Materials for this manuscript includes the following:

Tables S1-S21

**Supplementary Figures**


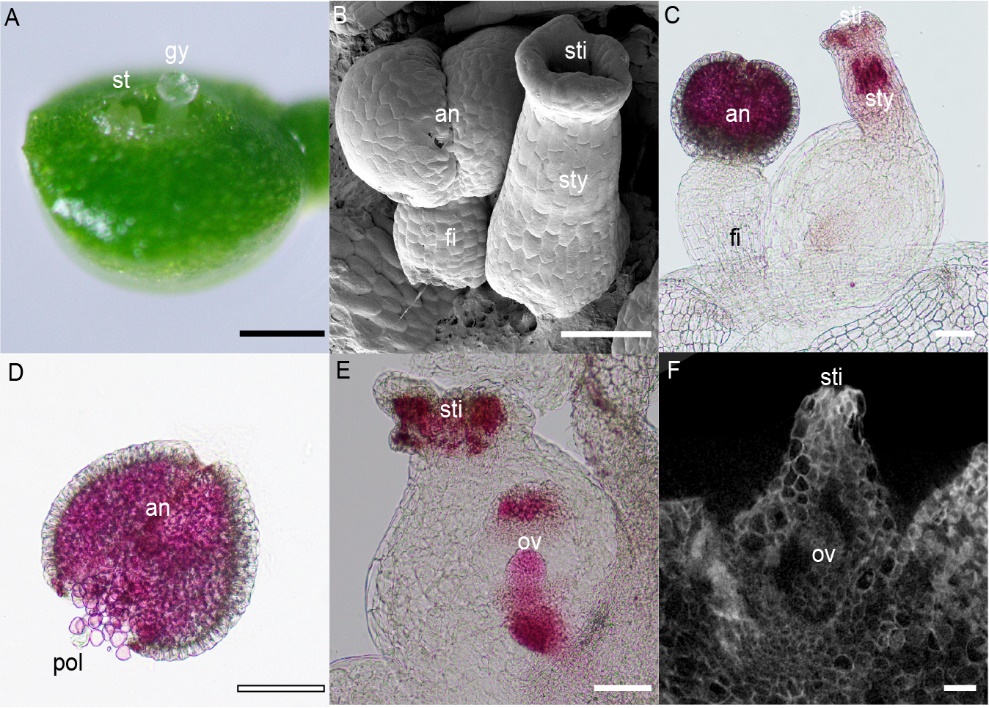


**Fig. S1. The *Wolffia australiana* floral organ structure.** The *Wolffia australiana* floral organ is shown in (**A**) to (**F**). Abbreviations: st, stamen; gy, gynoecium; an, anther; fi, filament; sti, stigma; sty, style; pol, pollen; ov, ovule. Bars: black = 1 mm, white = 100 μm.


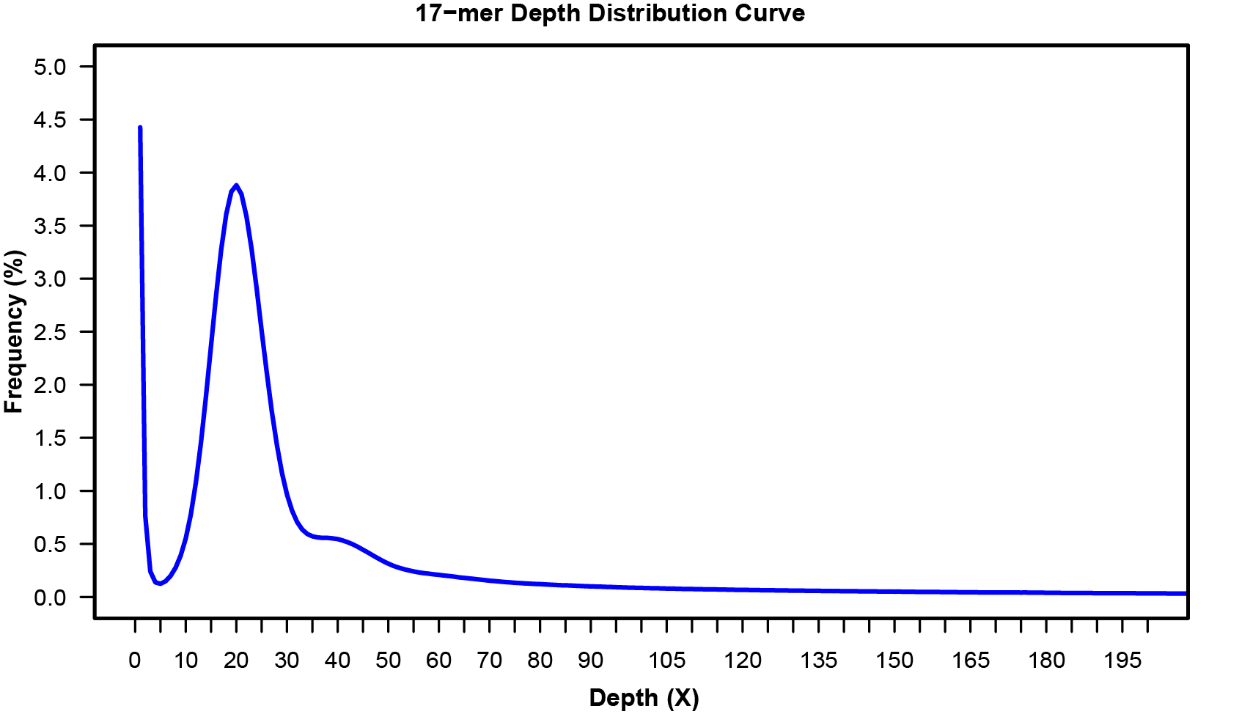


**Fig. S2.** The frequency distribution of 17-mer.


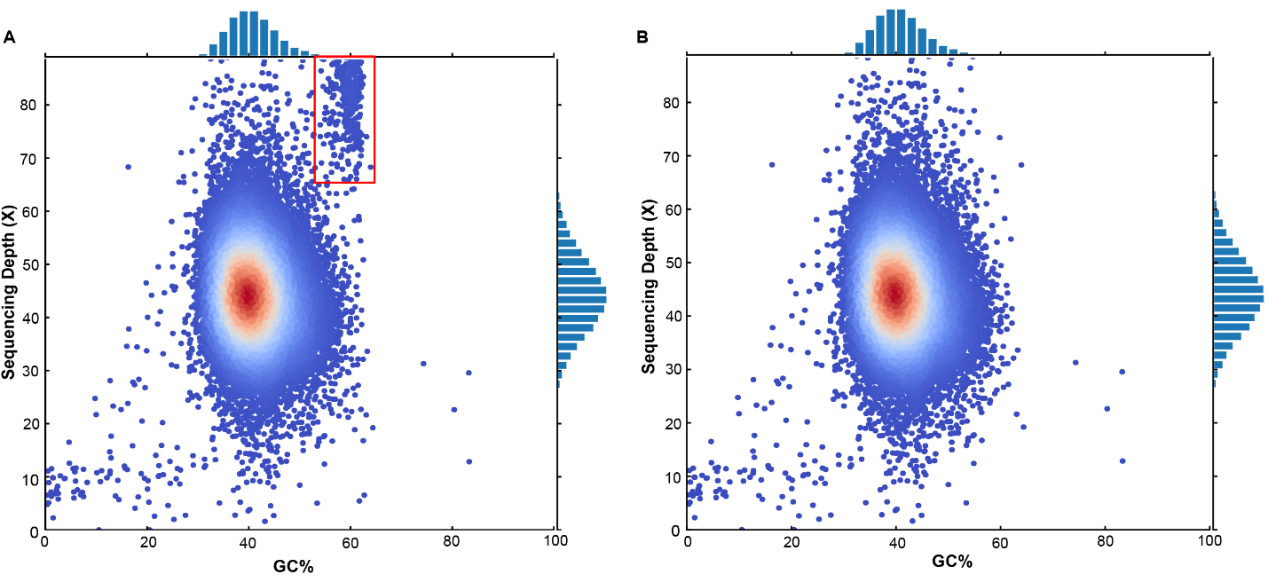


**Fig. S3.** The GC-Depth of *Wolffia australiana* genome based on the 10-kb bins. Panel (A) is the genome version G2, and panel (B) is the genome version G3. These bins of contaminative contigs are in the red frame.


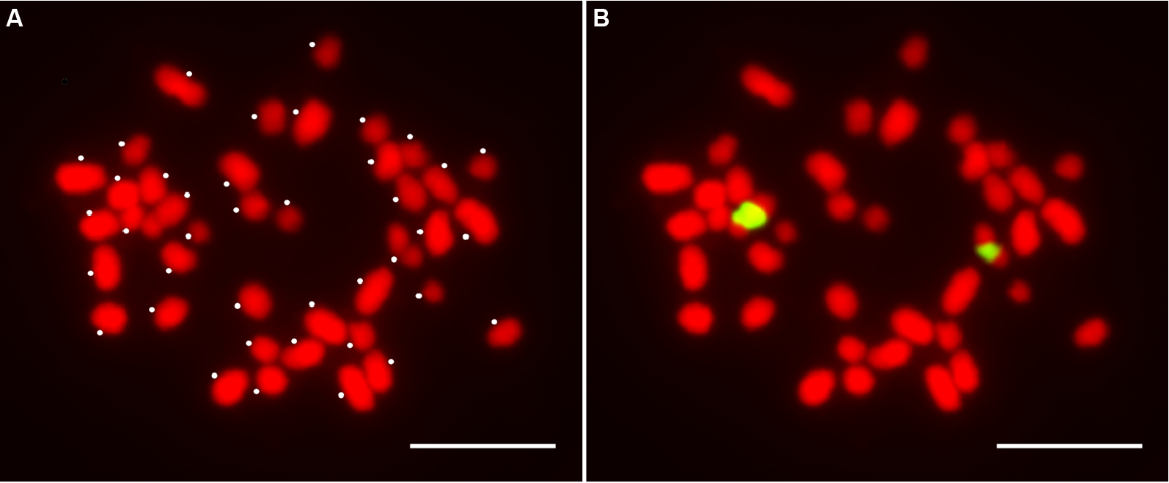


**Fig. S4.** Somatic metaphase chromosomes of *Wolffia Australiana*. Chromosomes (pseudocolored in red) were stained with 4′,6-diamidino-2-phenylindole (DAPI). (A) The white dot beside each chromosome indicates the individual chromosome, and the total chromosome number was 40. (B) The same cell probed with 45S rDNA showing a pair of chromosomes with 45S rDNA (pseudocolored in green) located in the middle of the chromosome. Bar = 5 μm.


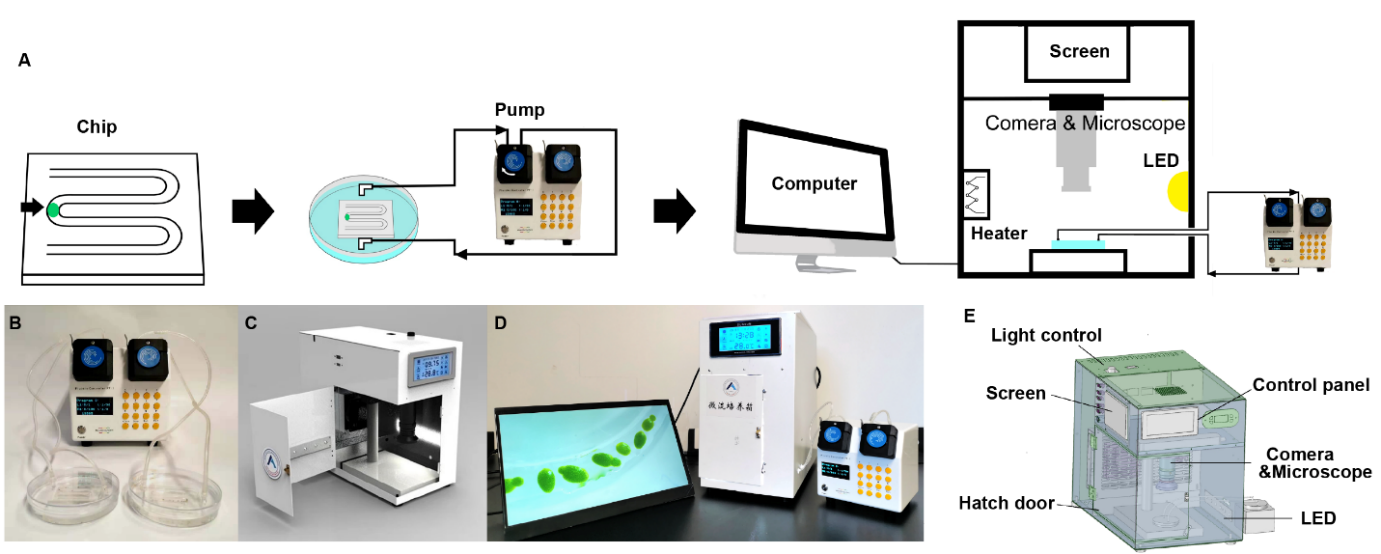


**Fig. S5.** Composition of the Plant-on-Chip Culture System. (A) Schematic structure of the system. (B) The chip and pump. (C) Design sketch of the incubator. (D) The PoC system. (E) The blueprint of the incubator.


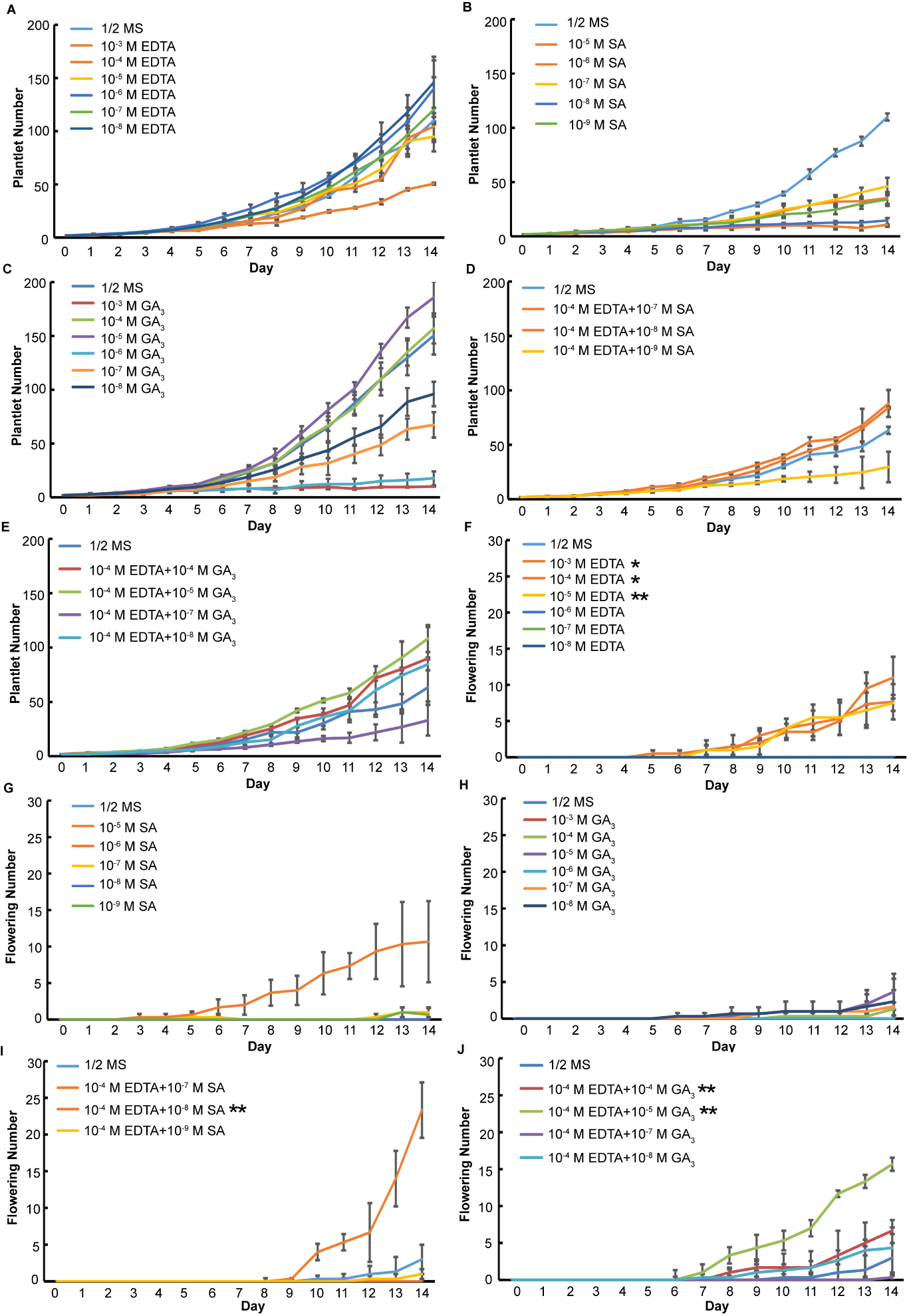


**Fig. S6.** The *Wolffia australiana* flowering rate upon various treatments. The *W. australiana* branching speed is shown in (A) to (E). The plantlet was grown on 1/2 MS medium with EDTA (A), SA (B), GA_3_ (C), EDTA+SA (D), or EDTA+GA_3_ (E) for 2 weeks. The flowering plantlet numbers of each group are shown in (F) to (J) (mean ± SEM, n = 3). **P* < 0.05, ***P* < 0.01, two-tailed Student’s *t*-test for all pairwise comparisons.


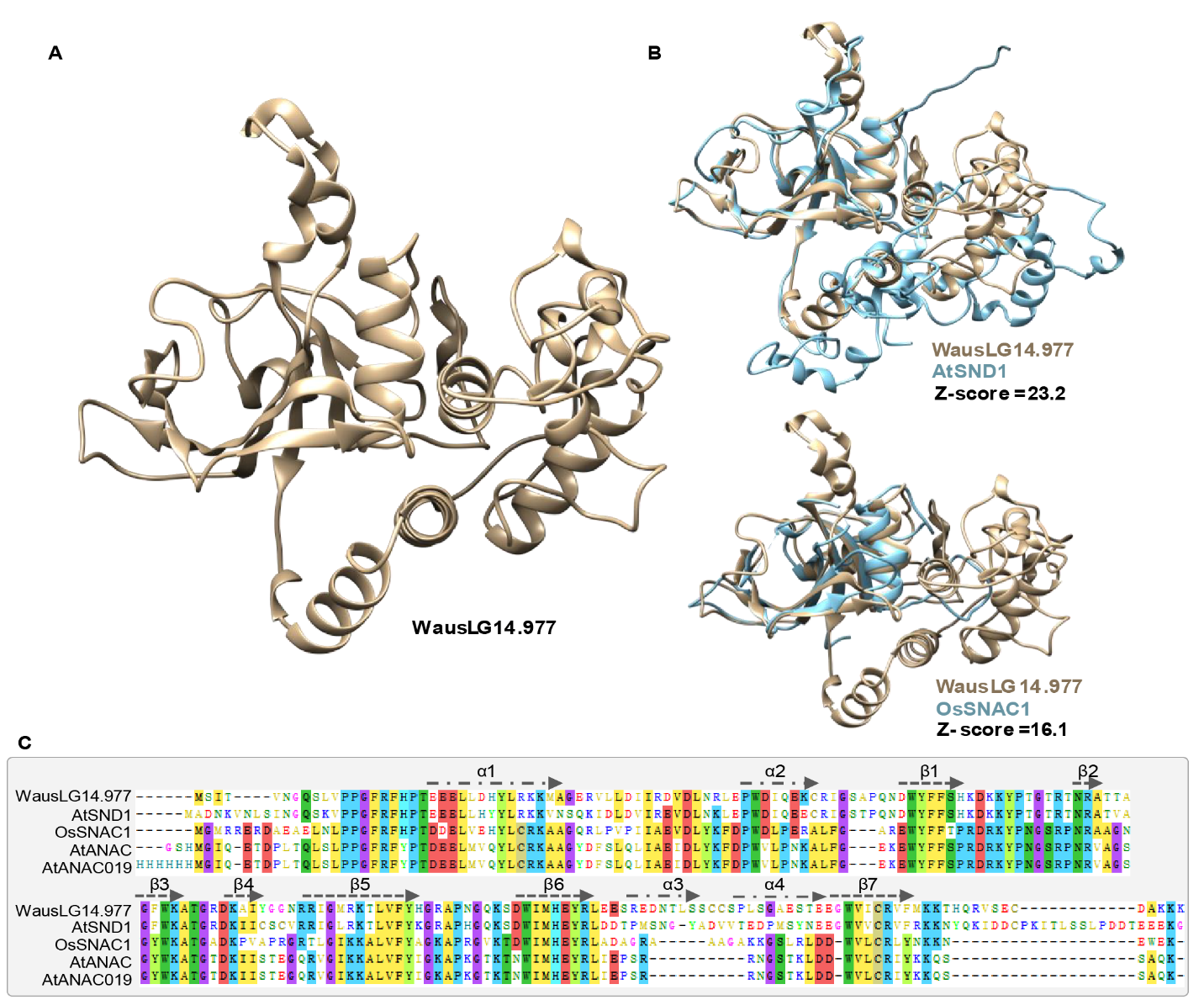


**Fig. S7.** Protein structure analyses of WausLG14.977 predicted using RoseTTAFold. (A) The 3D structure of WausLG14.977 predicted by using the RoseTTAFold server (https://robetta.bakerlab.org). (B) Structure comparison of WausLG14.977 with that of AtSND1 and OsSNAC1. Z-scores are indicated. (C) Sequence alignment of WausLG14.977 and the NAC homologs using ClustalW and ENDscript/ESPript. Z-scores of WausLG14.977 protein structure relative to those of Arabidopsis SND1 and rice SNAC1 were determined using the Dali server (https://ekhidna2.biocenter.helsinki.fi/dali).


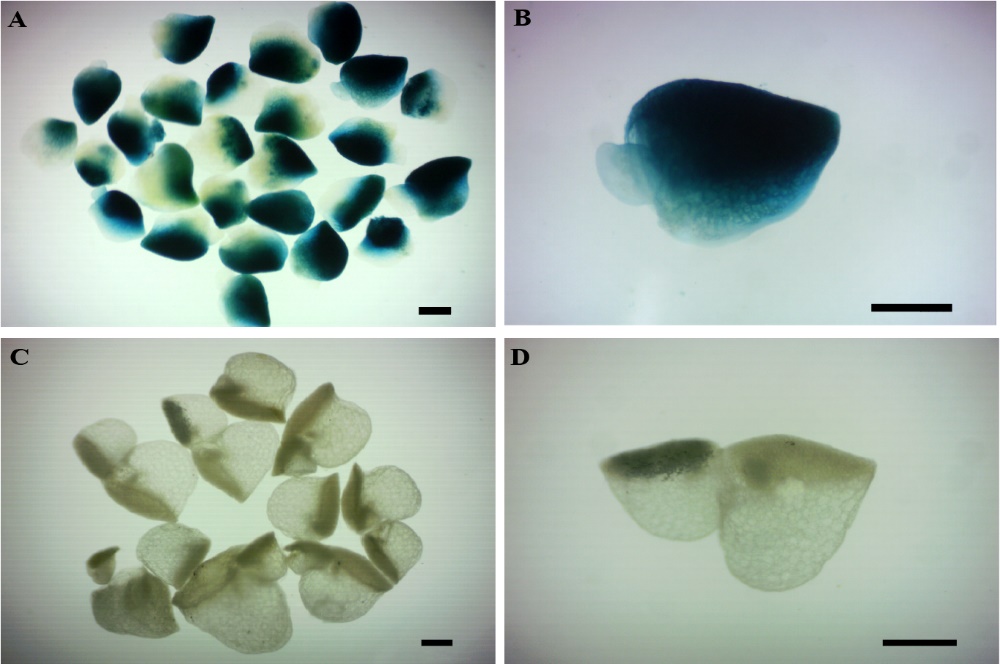


**Fig. S8.** Transgenic plantlets with GUS staining of *Wolffia australiana*. (A and B) GUS staining showing the expression of cytokinin in *W. australiana.* (C and D) GUS staining of Wolffia transformed by an empty vector. Bar = 500 μm.**Table S22. All reagents, data and software are listed below.**

| **REAGENT or RESOURCE** | **SOURCE** | **IDENTIFIER** |
| --- | --- | --- |
| **Chemicals, Peptides, and Recombinant Proteins** | | |
| Acetosyringone | Sigma-Aldrich | CAS 2478-38-8 |
| Cefotaxime | Sigma-Aldrich | CAS 64485-93-4 |
| Geneticin | Sigma-Aldrich | CAS 108321-42-2 |
| X-Gluc | Sigma-Aldrich | CAS 129541-41-9 |
| Silwet-77 | Coolaber | Cat# CS9791 |
| Peracetic acid | Aladdin | Cat# P299577 |
| Direct Red 23 | Sigma-Aldrich | Cat# 212490 |
| Trifluoroacetic acid | Sigma-Aldrich | Cat# T6508 |
| α-Amylase | Megazyme | Cat# E-BLAAM |
| Sodium borohydride | Sigma-Aldrich | Cat# 213462 |
| Sucrose | Sigma-Aldrich | Cat# S8501 |
| Glycerol | Sigma-Aldrich | Cat# G5516 |
| Ethyl acetate | J.T.Baker | Cat# 9282-03 |
| Anthrone | Sigma-Aldrich | Cat# 319899 |
| Acetic acid | J.T.Baker | Cat# 9515-03 |
| Glutaraldehyde | Sigma-Aldrich | Cat# G7651 |
| Paraformaldehyde | Sigma-Aldrich | Cat# P6148 |
| Formaldehyde | Sigma-Aldrich | Cat# 252549 |
| Glycine | Sigma-Aldrich | Cat# 241261 |
| DpnII | New England Biolabs | Cat# R0543S |
| Biotin-14-dCTP | Thermo Fisher Scientific | Cat# 19518018 |
| T4 DNA polymerase | New England Biolabs | Cat# M0203S |
| Cellulase | Sigma-Aldrich | Cat# C0615 |
| Pectinase | Sigma-Aldrich | Cat# P2611 |
| Trichloroacetaldehyde | LMAI Bio | Cat# LM008072 |
| TRIzol reagent | Invitrogen | Cat# 15596018 |
| CTAB | Sigma-Aldrich | Cat# H6269-100G |
| LiCl | Sigma-Aldrich | Cat# L9650 |
| Thiocarbohydrazide | Sigma-Aldrich | Cat# 223220 |
| Digoxigenin | Sigma-Aldrich | Cat# D9026 |
| Anti-digoxigenin-Rhodamine | Sigma-Aldrich | Cat# 11207750910 |
| Sodium borohydride | Sigma-Aldrich | Cat# 213462 |
| Acetic anhydride | Sinopharm | Cat# 10000318 |
| Anthrone | Sigma-Aldrich | Cat# 319899 |
| Ethyl acetate | Sigma-Aldrich | Cat# 270989 |
| Na_2_HPO_4_·12H_2_O | Sigma-Aldrich | Cat# 71649 |
| NaH_2_PO_4_·H_2_O | Sigma-Aldrich | Cat# 71507 |
| Osmium Tetroxide, 4% aqueous (OsO_4_) | Ted Pella | Cat# 18459 |
| Potassium ferrocyanide | Sigma-Aldrich | Cat# P3289 |
| 8-hydroxyquinoline | Sigma-Aldrich | Cat# 252565 |
| Methanol | Sigma-Aldrich | Cat# 1424109 |
| Nitric acid | Sinopharm | Cat# 10014518 |
| Sulfuric acid | Sigma-Aldrich | Cat# 339741 |
| Anti-fade solution | Vector Laboratories | Cat# H-1200-10 |
| Spurr’s resin | SPI Supplies | Cat# 02680-AB |
| Poly-T oligo-attached magnetic beads | New England Biolabs | Cat# E7490 |
| MS medium | Solarbio | Cat# M8521 |
| SH medium | Solarbio | Cat# LA8620 |
| GA_3_ | Aladdin | Cat# G105689 |
| SA | Sigma | Cat# S7401 |
| EDTA | Sigma | Cat# E4884 |
| PDSM | Momentive | Cat# RTV615 |
| Pump | MesoBioSystem | Cat# pp1 |
| Critical Commercial Assays | | |
| RNeasy® Plant Mini Kit | Qiagen | Cat# 74903 |
| Qubit RNA Assay Kit | Life Technologies | Cat# Q32852 |
| NEBNext Ultra^TM^ RNA Library Prep Kit Illumina | New England Biolabs | Cat# E7530L |
| NEBNext First Strand Synthesis Module | New England Biolabs | Cat# E7525L |
| NEBNext Ultra II Non-Directional RNA Second Strand Synthesis Module | New England Biolabs | Cat# E6111L |
| Bionano Prep DLS Labeling DNA Kit | Bionano Genomics | Cat# 80005 |
| Bionano Prep DLS Labeling DNA Kit | Bionano Genomics | Cat# 80005 |
| KAPA Hyper Prep Kit | KAPA Biosystems | Cat# KK8504 |
| TruSeq RNA Library Preparation Kit | Illumina | Cat# RS-122-2001/2002 |
| M-MuLV reverse transcriptase | New England Biolabs | Cat# M0253L |
| USER enzyme | New England Biolabs | Cat# M5505L |
| AMPure XP system | Beckman Coulter | Cat# A63882 |
| Single-Cell Library Prep Set | MGI | Cat# 1000021082 |
| Ligation Sequencing Kit | Nanopore store | Cat# SQK-LSK109 |
| **Deposited Data** |  |  |
| *Wolffia australiana 7733* (Waus) | *Wolffia australiana 7733* (Waus) genome, this study | NCBI Genome: CP092600-CP092619, 20 chromosomes. NCBI sequence read archive (SRA): PRJNA808652, Nanopore; PRJNA808655, Illumina genome; PRJNA808685, Hi-C; PRJNA808734, BioNano; PRJNA808736, RNA-seq for genome; PRJNA808739, single-plant RNA-seq; PRJNA809022 single-nucleus RNA-seq. NCBI Supplementary Files: SUPPF_0000004267, BioNano. <http://wolffiapond.net/> |
| *Chlamydomonas reinhardtii* | *Chlamydomonas reinhardtii* genome | <https://www.ncbi.nlm.nih.gov/genome/?term=Chlamydomonas+reinhardtii+> |
| *Azolla filiculoides Lam.* | *Azolla filiculoides Lam.* genome | <https://www.fernbase.org/?tdsourcetag=s_pcqq_aiomsg> |
| *Physcomitrella patens* | *Physcomitrella patens* genome | <https://www.ncbi.nlm.nih.gov/genome/?term=Physcomitrella+patens> |
| *Amborella trichopoda* | *Amborella trichopoda* genome | <https://www.ncbi.nlm.nih.gov/genome/?term=Amborella+trichopoda> |
| *Oryza sativa L.* | *Oryza sativa L*. genome | <https://www.ncbi.nlm.nih.gov/genome/?term=Oryza+sativa> |
| *Spirodela polyrhiza* | *Spirodela polyrhiza* genome | <https://www.ncbi.nlm.nih.gov/genome/?term=Spirodela+polyrhiza> |
| *Spirodela polyrhiza 9509* | *Spirodela polyrhiza 9509* genome | <ftp://ftp.lemna.org/spirodela_polyrhiza_9509/> |
| *Lemna minor 8627* | *Lemna minor 8627* genome | <ftp://ftp.lemna.org/lemna_minor_8627/> |
| *Lemna gibba 7742a* | *Lemna gibba 7742a* genome | <ftp://ftp.lemna.org/lemna_gibba_7742a/> |
| *Wolffia australiana 8730* | *Wolffia australiana 8730* genome | <ftp://ftp.lemna.org/wolffia_australiana_8730/> |
| *Colocasia esculenta* | *Colocasia esculenta* genome | <https://www.ncbi.nlm.nih.gov/genome/?term=Colocasia+esculenta> |
| *Zostera marina* | *Zostera marina* genome | <https://www.ncbi.nlm.nih.gov/genome/?term=Zostera+marina> |
| *Elaeis guineensis* | *Elaeis guineensis* genome | <https://www.ncbi.nlm.nih.gov/genome/?term=Elaeis+guineensis> |
| *Arabidopsis thaliana* | *Arabidopsis thaliana* genome | [https://www.ncbi.nlm.nih.gov/genome/?term=Arabidopsis+thaliana)](https://www.ncbi.nlm.nih.gov/genome/?term=Arabidopsis+thaliana) |
| *Nymphaea colorata* | *Nymphaea colorata* genome | <https://www.ncbi.nlm.nih.gov/genome/69117> |
| *Utricularia gibba* | *Utricularia gibba* genome | <https://www.ncbi.nlm.nih.gov/genome/?term=Utricularia+gibba> |
| *Cuscuta australis* | *Cuscuta australis* genome | <https://www.ncbi.nlm.nih.gov/genome/?term=Cuscuta+australis> |
| *Musa acuminata* | *Musa acuminata* genome | <http://plants.ensembl.org/Musa_acuminata/Info/Index> |
| Kyoto Encyclopedia of Genes and Genomes | Biological organisam pathways database | <https://www.kegg.jp/> |
| Eukaryotic orthgene Groups of protein | Eukaryotic coding proteins' phylogenetic classification database | <http://genome.jgi-psf.org/help/kogbrowser.jsf> |
| SwissProt | Validated rigorously de-redundant protein sequences database | <https://web.expasy.org/docs/swiss-prot_guideline.html> |
| Non +72:75redundant Protein sequence databases | Non redundant Protein sequence database | <ftp://ftp.ncbi.nlm.nih.gov/blast/db/> |
| PDB sequence data | (*52, 53*) | <ftp://ftp.wwpdb.org/pub/pdb/derived_data/pdb_seqres.txt> |
| SCOPE 2.08-stable | (*55, 56*) | <https://scop.berkeley.edu/downloads/ver=2.08> |
| AlphaFold Protein Structure Database: proteome-wide predictions | (*31*) | <https://www.alphafold.ebi.ac.uk/download#proteomes-section> |
| **Experimental Models: Organisms/Strains** | | |
| *Wolffia australiana* | Institute of Hydrobiology, CAS | Accession: wa7733 |
| **Oligonucleotides** |  |  |
| Primer: LG14-GW-F. GGGGACAAGTTTGTACAAAAAAGCAGGCTACATGAGCATCACGGTCAACG | This study | N/A |
| Primer: LG14-1-R. TGACCCAATCCTGCACTTCTCTTGAATGTCCCAAGGCTCAA | This study | N/A |
| Primer: LG14-2-F.AGAAGTGCAGGATTGGGTCA | This study | N/A |
| Primer: LG14-2-R.CCGGAAAGCGGACTACAACAACTGGAAAGGGTGTTATCTTCT | This study | N/A |
| Primer: LG14-3-F.TGTTGTAGTCCGCTTTCCGG | This study | N/A |
| Primer: LG14-GW-R.GGGGACCACTTTGTACAAGAAAGCTGGGTACTATCCACCGGGTGTAGA | This study | N/A |
| Recombinant DNA |  |  |
| pCAMBIA1300-PacI | (*60*) | Addgene ID: 44183 |
| pCAMBIA1300-WausLG14.977 | This study | N/A |
| **Software and Algorithms** |  |  |
| AlphaFold v2.0.1 | (*30*) | <https://github.com/deepmind/alphafold> |
| MMseqs2 Release 13-45111 | (*32*) | <https://github.com/soedinglab/MMseqs2> |
| DaliLite.v5 | (*54*) | <http://ekhidna2.biocenter.helsinki.fi/dali/> |
| Pandas 1.3.3 | (*61*) | <https://zenodo.org/record/5501881#.YckwaGBBxhE> |
| STAR V2.7.4a | (*62*) |  |
| PISA V0.8.2 | (*63*) | <https://github.com/shiquan/pisa> |
| Seurat V3.2.1 | (*64*) | <https://satijalab.org/seurat> |
| Seurat V3.2.2 | (*64*) | <https://satijalab.org/seurat> |
| Seurat V3.6.3 | (*64*) | <https://satijalab.org/seurat> |
| MEGA6 | (*50*) | <https://www.megasoftware.net/> |
| PLAZA | (*65*) | <https://bioinformatics.psb.ugent.be/plaza/> |
| ClustalW | (*66*) | <https://www.clustal.org/> |
| ENDscript/ESPript | (*67*) | [https://endscript.ibcp.fr](https://endscript.ibcp.fr/) |
| RoseTTAFold server | (*51*) | <https://robetta.bakerlab.org/> |
| UCSF Chimera | (*68*) | <https://www.rbvi.ucsf.edu/chimera> |
| RCSB PDB database | (*53*) | <https://www.rcsb.org/> |
| Dali server | (*54*) | <https://ekhidna2.biocenter.helsinki.fi/dali> |
| BWA 0.7.12-r1039 | (*69*) | <https://sourceforge.net/projects/bio-bwa/> |
| Hmmer 3.0 | (*70*) | <http://www.hmmer.org/download.html> |
| FIMO 4.11.4 | (*71*) | <https://meme-suite.org/meme/doc/fimo.html> |
| Igraph 1.2.7 | R package | <https://igraph.org/r/> |
| BLAST v2.9 | Homolougs alignments for nucleotide sequences | <https://blast.ncbi.nlm.nih.gov/Blast.cgi> |
| NextDenovo v2.0-beta.1 | Self-error correction software of the Nanopore original data | <https://github.com/Nextomics/NextDenovo/> |
| NextGraph v2.0-beta.1 | Genome contig assembly software | <https://github.com/Nextomics/NextDenovo/> |
| Minimap2 r41 | Sequence alignment software | <https://github.com/lh3/minimap2> |
| Racon v1.4.3 | Error correction software | <https://github.com/isovic/racon> |
| Fastp v0.19.4 | Quality control software for Illumina raw data | <https://github.com/OpenGene/fastp> |
| BWA 0.7.12-r1039 | Sequence alignment of genomic data on Illumina platform | <http://bio-bwa.sourceforge.net/> |
| NextPolish v1.0.5 | Error correction software of NGS data | <https://github.com/Nextomics/NextPolish.git> |
| SAMtools v1.4 | Analysis tool for sam and bam files | <https://github.com/samtools/samtools> |
| BCFtools v1.8.0 | Analysis tool for vcf files | <https://github.com/samtools/bcftools> |
| HISAT2 v2.1 | Sequence alignment of transcriptome data on Illumina platform | <https://daehwankimlab.github.io/hisat2/> |
| BUSCO v3.1.0 | Genome completeness evaluation software by using OrthoDB database | <https://busco.ezlab.org/> |
| CEGMA v2 | Genome completeness evaluation software by using core genes | <https://github.com/KorfLab/CEGMA_v2/> |
| Bowtie2 v2.3.2 | Sequence alignment of sequences on Illumina platform | <http://bowtie-bio.sourceforge.net/bowtie2/index.shtml> |
| Bionano Solve™ data analysis software | Bionano optical genome mapping | <https://bionanogenomics.com/products/bionano-data-solutions/> |
| LACHESIS | Hi-C scaffolding software | <https://github.com/shendurelab/LACHESIS> |
| GMATA v2.2 | Simple sequence repeats identification software | <https://sourceforge.net/projects/gmata/> |
| Tandem Repeats Finder v4.07b | Tandem repeat sequences identification software | <https://tandem.bu.edu/trf/trf.download.html> |
| LTR_finder v1.07 | Long terminal repeat retrotransposons identification software | <http://tlife.fudan.edu.cn/tlife/ltr_finder/help/single.html> |
| LTR_harverst v1.5.10 | Long terminal repeat retrotransposons identification software | <http://genometools.org/tools/gt_ltrharvest.html> |
| LTR_retriver v1.8.0 | Long terminal repeat library construction software | <https://github.com/oushujun/LTR_retriever.git> |
| MITE-Hunter v11-2011 | Miniature inverted transposable elements identification software | <http://target.iplantcollaborative.org/mite_hunter.html> |
| RepeatModeler v1.0.11 | Genome masking and novel transposable elements identification software | <https://github.com/Dfam-consortium/RepeatModeler> |
| RepeatMasker v1.331 | Repetitive sequences identification software | <http://www.repeatmasker.org/> |
| GeMoMa v1.6.1 | Gene prediction software based on homolog proteins | <http://www.jstacs.de/index.php/GeMoMa> |
| StringTie v1.3.3d | Gene prediction software based on transcriptome data on Illumina platform | <https://ccb.jhu.edu/software/stringtie/> |
| PASA v2.3.3 | Transcriptome assembly software | <https://github.com/PASApipeline/PASApipeline/wiki> |
| TransDecoder | Protein-coding region prediction software | <https://github.com/TransDecoder/TransDecoder/wiki> |
| AUGUSTUS v3.3.1 | *De novo* gene prediction software | <http://augustus.gobics.de/> |
| EVidenceModeler v1.1.1 | Software combines ab intio gene predictions and protein and transcript alignments into weighted consensus gene structures | <https://evidencemodeler.github.io/> |
| Infernal v1.1.2 | rRNA, snRNA and miRNA prediction software | <http://eddylab.org/infernal/> |
| tRNAscan-SE v2.0 | tRNAs prediction software | <http://lowelab.ucsc.edu/tRNAscan-SE/> |
| RNAmmer v1.2 | rRNAs prediction software | <http://www.cbs.dtu.dk/services/RNAmmer/> |
| OrthoMCL v2.0.9 | Homolougs alignments for protein sequences | <https://orthomcl.org/orthomcl/> |
| MAFFT v7.313 | Multiple sequence alignments of gene families | <https://myhits.sib.swiss/cgi-bin/mafft> |
| Gblocks v0.91b | Protein multiple sequence alignment software | <http://molevol.local/castresana/Gblocks/> |
| RAxML v8.2.10 | Phylogenetic tree construction software | <https://github.com/amkozlov/raxml-ng> |
| Figtree v1.4.4 | Phylogenetic tree editing software | <http://tree.bio.ed.ac.uk/software/figtree/> |
| CAFE v4.2.1 | Gene family contraction and expansion analysis software | <https://github.com/hahnlab/CAFE> |
| ClusterProfiler | GO and KEGG enrichment software | <https://github.com/YuLab-SMU/clusterProfiler> |
| McScanX | Collinear block computing software within or between genomes | <http://chibba.pgml.uga.edu/mcscan2/> |
| KaKs_Calculator v2.0 | Synonymous substitution rate calculation software | <https://sourceforge.net/projects/kakscalculator2/> |
| DESeq2 package | Differential expression genes calculation software | <http://bioconductor.org/packages/release/bioc/html/DESeq2.html> |
| Interproscan v5.32-71.0 | Gene Ontology analysis software | <https://github.com/ebi-pf-team/interproscan/wiki> |
| **Other** |  |  |
| SEM | Thermo Fisher Scientific | Helios NanoLab G3 UC |
| Cryo-Stage | Quorum Technologies | PP3010T |
| MicroCT | Zeiss | Xradia Context |
| MicroCT | Bruker | SkyScan 1272 |
| Ultramicrotome | Leica Microsystem | UC7 |
| TEM | Jeol | JEM-1400 |
| Qubit 2.0 fluorometer | Life Technologies | REQ32866 |
| Agilent Bioanalyzer 2100 system | Agilent | G2939BA |
| Promethion | Oxford Nanopore Technologies |  |
| Illumina NovaSeq 6000 platform | Illumina | Cat# 20012850 |

**References in the Supplementary Material**

60. **Tang G, et al.** 2012. Construction of short tandem target mimic (STTM) to block the functions of plant and animal microRNAs. *Methods* 58:118–125.

61. **McKinney W**. 2010. Data structures for statistical computing in Python. Proceedings of the 9th Python in Science Conference. 51–56.

62. **Dobin A, et al.** 2013. STAR: ultrafast universal RNA-seq aligner. *Bioinformatics* 29:15–21.

63. **Mallia A, Siedlaczek M, Mackenzie J, Suel T**. 2019. PISA: performant indexes and search for academia. *CEUR Workshop Proc*. 2409:50–56.

64. **Stuart T, et al.** 2019. Comprehensive integration of single-cell data. *Cell* 177:1888–1902.e1821.

65. **Van Bel M, et al.** 2018. PLAZA 4.0: an integrative resource for functional, evolutionary and comparative plant genomics. *Nucleic Acids Res*. 46:D1190–D1196.

66. **Larkin MA, et al.** 2007. Clustal W and Clustal X version 2.0. *Bioinformatics* 23:2947–2948.

67. **Robert X, Gouet P.** 2014. Deciphering key features in protein structures with the new ENDscript server. *Nucleic Acids Res*. 42:W320–W324.

68. **Pettersen EF, et al.** 2004. UCSF Chimera—a visualization system for exploratory research and analysis. *J Comput Chem*. 25:1605–1612.

69. **Li H, Durbin R**. 2009. Fast and accurate short read alignment with Burrows–Wheeler transform. *Bioinformatics* 25:1754–1760.

70. **Mistry J, Finn RD, Eddy SR, Bateman A, Punta M**. 2013. Challenges in homology search: HMMER3 and convergent evolution of coiled-coil regions. *Nucleic Acids Res*. 41:e121.

71. **Grant CE, Bailey TL, Noble WS**. 2011. FIMO: scanning for occurrences of a given motif. *Bioinformatics* 27:1017–1018.
